# Green Chemistry-Assisted Synthesis of Metal Nanoparticles and Fabrication of Microstructurally Engineered Conductive and Endurable M^0^@PEO Functional Films

**DOI:** 10.1021/acsomega.5c03323

**Published:** 2025-08-19

**Authors:** Anamika Das, Raktima Chatterjee, Shinjini Sarkar, Grishma Ninave, Debosreeta Bose, Amit Kumar Dutta, Satarupa Biswas, Moumita Mukherjee, Ragavendran Venkatesan, Rahul Majee, Saumya Dasgupta, Jayanta Mukhopadhyay, Madhumita Mukhopadhyay

**Affiliations:** † Department of Physics, Acharya Prafulla Chandra College, New Barrackpore, Kolkata 700131, West Bengal, India; ‡ Department of Physics, 502852 Adamas University, Kolkata–Barrackpore–Barasat Road, Kolkata 700126, West Bengal, India; § Specialty Glass Division, 30148 CSIR-Central Glass & Ceramic Research Institute, Jadavpur, Kolkata 700032, West Bengal, India; ∥ Academy of Scientific and Innovative Research (AcSIR), Ghaziabad 201002, Uttar Pradesh, India; ⊥ Department of Chemistry, Amity Institute of Applied Sciences (AIAS), 530170 Amity University, Kolkata 700156, West Bengal, India; # Department of Chemistry, 530017 Bangabasi Morning College, 19 Raj Kumar Chakraborty Sarani, Kolkata 700009, West Bengal, India; ∇ Department of Physics, 29862 Rajalakshmi Engineering College, Thandalam, Chennai 602105, Tamil Nadu, India; ○ School of Chemistry, 7486 University of St Andrews, St Andrews, Fife KY16 9ST, United Kingdom; ◆ Energy Materials & Devices Division, CSIR-Central Glass and Ceramic Research Institute, Kolkata 700032, West Bengal, India

## Abstract

The present research reports the synthesis of poly­[ethylene oxide]-based composite films (500 μm) containing metal nanoparticles (NPs) [Ag^0^ (*d*_p_ ∼ 6 nm), Cu^0^ (*d*_p_ ∼ 25 nm), and Fe^0^ (*d*_p_ ∼ 35 nm)] as the mobile phase. The novelty of the study is in the corroboration of a plausible mechanism for the generation of metal NPs through green synthesis using herbal extracts of *Camellia sinensis* (Tea) and *Azadirachta indica* (Neem). Density functional theory (DFT) is used to optimize the phytoreductants present in both biosources, wherein the reducing and/or stabilizing functional entities are primarily hydroxyl groups (−OH). The transition energy (band gap, Δ*E*_|LUMO–HOMO|_) is found to be minimum for Epicatechin gallate (1.05 eV, tea) and Sitosterol (0.58 eV, neem), which could act as potent phytoreductants for initiating a redox reaction, followed by subsequent capping through secondary bond formation. Upon increasing the loading of metal NPs from 1 to 7 wt %, the ionic conductivity of a PEO composite increases (0.1 S·cm^–1^) for PAg_N_ (N: neem). With a subsequent increase in loading (10 wt %), the crystalline region within PEO is enhanced (≥83% using DSC), which restricts the ion migration and lowers the charge storage capacity, as studied using dielectric constants and complex relaxation processes (EIS and DRT). Among all of the compositions, PCu_N_ is observed to exhibit negligible performance deterioration (Δtan δ for 18,500 h → 0). However, PAg system(s) are good ion conductors with significant dielectric nature, but they suffer from particle ripening. Hence, metal NPs, which functionalize PEO films, could be effectively synthesized using a green synthesis route and applied as a solid electrolyte for device application.

## Graphical abstract

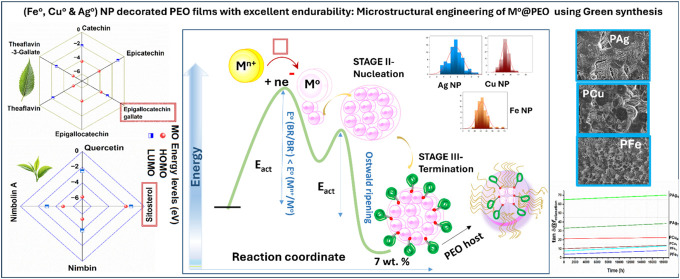

## 1 Introduction

Polymer-based composites have received significant attention due to their multivariant applications in the field of photonics, energy harvesting devices, point of care application(s), catalysis, etc.^1–3^ In this regard, polymer-based nanocomposite films decorated with metal/metal oxide nanoparticles have achieved special attention, owing to their functionality. Jayakumar et al. and many other researchers have reported the significance of ZnO and Ag nanoparticle encapsulation within biopolymers such as starch, chitosan, etc., which enable their utilization toward film formulation. These antioxidants offer significant technology for advancement in food packaging, antioxidants, drug carriers for antimicroscopic aids,^4–7^ etc. Blending such polymers with inorganic components through a chemical synthesis route has certain intrinsic shortcomings that restrict their long-term performance.^8,9^ Dimensional optimization of the synthesized metal/metal oxide nanoparticles is critical. In addition, the developed nanoparticles synthesized using chemical means are susceptible to agglomeration, which limits their functionality. Therefore, stabilization of such nanoparticles and avoiding chemical means for their synthesis (including significant cost and biohazard) are urgent areas of research.^8^ Jamkhande et al. reviewed the associated disadvantages of synthesis routes, namely, physical and chemical vapor deposition, hydrothermal method, spray pyrolysis, laser ablation, etc., toward the production of metal/metal oxide nanoparticles, which are termed as non-green techniques.^9^ Consequently, green synthesis routes involving plant extracts/micro-organisms are beneficial in terms of cost-effectiveness, safety, and having dual activity of acting as reducing and capping agents for the synthesized nanoparticles.^10,11^ Furthermore, polymers (host) can screen the growth of synthesized nanoparticles and inhibit the in situ sintering ability.^12^ The antioxidant properties of utilized phytoextracts can also be improved upon a suitable combination with metallic nanoparticles within a polymeric host matrix through synergistic influence. Hameed et al.^13^ reported an excellent swelling rate of nanofibers composed of biopolymers and neem extract with sustained release of phytonutrients, showing significant antifungal and antioxidant properties. Abdelghany et al.^7^ employed polymer nanocomposites with Au NPs as effective electrochemical and photoelectrical devices. Involvement of *Ricinus communis* has improved the overall electrical conductivity of polymer–Ag NPs from 7.62 × 10^–11^ to 2.04 × 10^–8^ S·cm^–1^ at 353 K with a much reduced activation barrier. Green synthesis of Cu NPs was reported by Pozdnyakov et al.,^14^ which contributes toward nontoxic hydrophilic antiseptics and antimicrobial components upon inclusion within poly-N-vinyl imidazole (Mw 23.5 kDa, PDI: 1.28). As-synthesized poly­(glycidyl methacrylate) decorated with Ag NPs in a raspberry-like fashion was reported by Chen et al.,^15^ which is suitable for the application in biomedicine, photonics, and electronics. *Acca sellowiana* leaves were employed by Sganzerla et al. for the biosynthesis of Ag NPs and were trapped with poly­[ethylene oxide],^16^ which acts as a food conservation and packaging material with a cost-effective processing route. Selection of a polymer host is also important in terms of application, which dictates the specificity of metal NPs and the bioreagent. Recently, Munir et al.^17^ explored the possibility of nonconducting and polyelectrolyte host(s) that uplift the efficiency of energy storage devices (sodium-ion batteries). Poly­(ethylene oxide) (PEO), poly­(vinyl alcohol) (PVA), polyacrylonitrile (PAN), etc., are some of the good candidates for the same. In contrast, arginine, chitosan, polysaccharides, poly­(glycolic acid), etc., are opted as hosts for application in polymeric drug delivery systems.^18,19^ Paquet et al. suggested the use of polystyrene (PS), poly­(methyl methacrylate) (PMMA), cyclic olefin polymers (COPs), etc., for photonics, since they satisfy the criteria of having optical clarity, compatible refractive index, etc.^20^ The authors have earlier reported the admirable functionality of PEO with inorganic salts such as NH_4_I, KBr, etc., along with their solvent sorption studies (non-Fickian diffusion behavior) for application as a solid polymer electrolyte.^21–24^ The addition of a nonconducting filler and γ irradiation on PEO are also studied to have a significant influence on the structure–property relation.^25–28^ The selection of the host polymer is based on salient features such as biodegradability, water solubility, wide miscible traits with variable metal salts, and compatible mechanical properties with state-of-the-art electrode materials applicable for solid-state devices.^29^

In the present investigation, we tried to address the long-term usability of PEO composite films having nanosized metals as mobile carriers. The present research reports the functionalization of the PEO host with metal nanoparticles (NP), namely, Fe^0^, Cu^0^, and Ag^0^, synthesized using phytoreductants from herbs such as *Camellia sinensis* (Tea) and *Azadirachta indica* (Neem). The novelty of the study aims at the prediction of a plausible mechanism for green synthesis of metal NPs using herbal extracts. The salient novelties of the present research are highlighted in Table S1 along with comparative prior art studies [S1–S6]. The phytochemicals present in the mentioned herbs are listed in Table S2,^30–35^ which provides an insight into the nature of reducing agents (along with capping species) that interact with the metal salts and stay within the composite polymer film. The synthesized PEO-based nanocomposites are analyzed to study ion conductivity and dielectric properties as a function of applied frequency using the electrochemical impedance (EIS) technique, and the results are correlated with morphological studies. Furthermore, the developed PEO films exhibit long-term (18,500 h) performance, wherein the ion conduction and dielectric storage could be tailored as a function of metal NPs using a specific selection of phytoreductants and capping agents. An attempt has been made to study the reactivity pattern of selective phytoreductants of tea and neem using density functional theory (DFT), based on which a temperature-dependent redox reaction is undertaken. DFT is employed to preselect the energetically favorable phytoreductants for metal ions such as Ag^+^, Cu^2+^, and Fe^2+^. A study on the long-term endurability of PEO nanocomposite films and its correlation with preselected phytoreductants using DFT is reported for the first time, as per the author’s knowledge.

## 2 Materials and Methods

### 2.1 Synthesis of Metal Nanoparticles and Analysis

All of the reagents and metal salts were of AR grade, purchased from Sigma-Aldrich, and used without further purification. The *Camellia sinensis* (Tea) and *Azadirachta indica* (Neem) were collected and washed with distilled water. Leaves were finely chopped after drying, and ∼10 g were added to 100 mL of distilled water and stirred at 60 °C for 1 h. The mixture was cooled and filtered for a single run with Whatman paper No. 1 filter paper (pore size ∼10 μm). The obtained filtrate was collected and stored in a refrigerator for further use. A refrigeration temperature of 4 °C was optimized for the storage of the extract, which could be used for a week. 1 × 10^–3^ M aqueous solutions of silver nitrate, cupric nitrate, and ferrous sulfate were separately prepared in 250 mL Erlenmeyer flasks. 5 mL of the extract (Tea and Neem as per experiment) was added to 95 mL of 1 mM metal solution at room temperature (25 °C). The rate of the reaction was studied by varying the temperature of the reaction mixture between 25 and 40 °C. Due to the slightly sluggish reaction kinetics of tea extract with AgNO_3_, a higher temperature of 90 °C was studied as well. The respective color changes upon saturation of the reaction mixture are shown in Figure 1. A reaction that responds within 2 min of the addition of reactants is termed a kinetically labile reaction, whereas if the first response is obtained after 4–5 h, it is known as moderately labile. The absence of any visible reactivity is named an inert reaction (Figure 2). In the case of AgNO_3_ solution, the reactions were carried out in the dark to avoid photoactivation. Post saturation of the reaction mixture (irrespective of the applied temperature), the reaction mixture was sonicated at 20 kHz frequency to enable precipitation of the developed product and was subjected to vacuum filtration (a mini vacuum pump fitted to a Buchner funnel) prior to drying. A UV–vis spectrophotometer (Hitachi U-2910 spectrophotometer) was used to characterize the optical spectra of the reaction mixture at variable time intervals to study the formation of metal nanoparticles. The nanoparticles’ size and the surface morphology were studied by a scanning electron microscope (SEM FEIC-QUO-35357-0614 with a Bruker Quantax 100). The histograms corresponding to the particle size distribution of the metal nanoparticles are plotted using ImageJ software.

**Figure 1:**
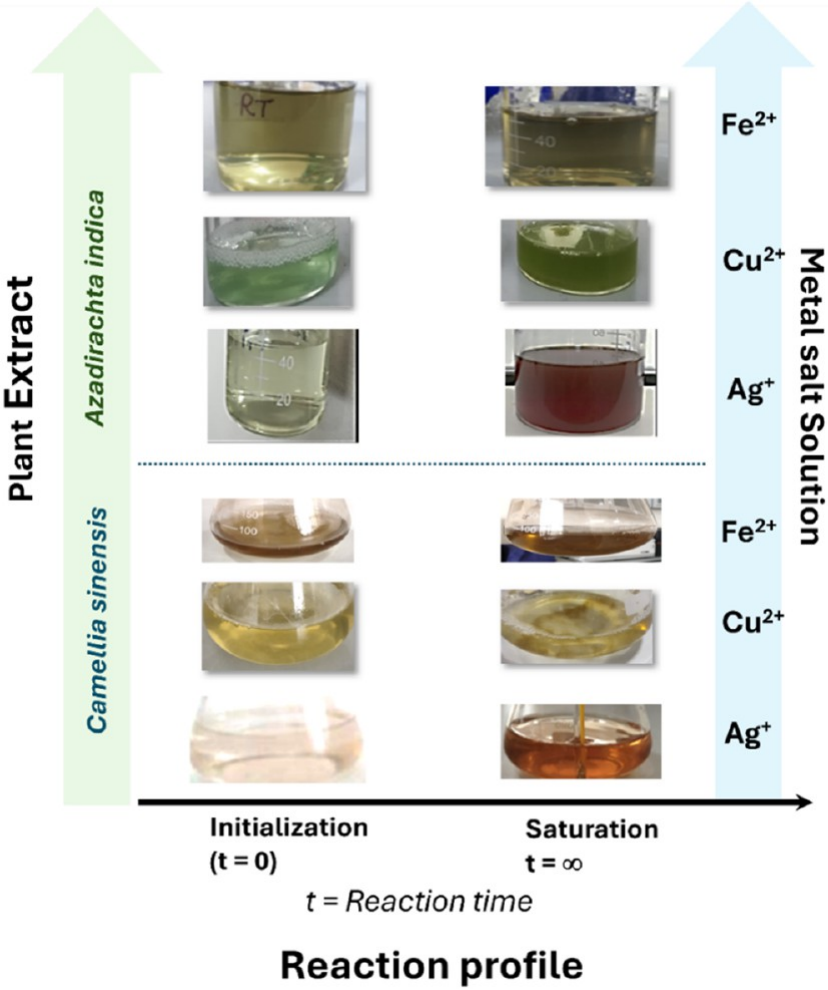
Colorimetric study of the reaction profile for plant extract and metal salt solutions at the initialization and saturation tenure.

**Figure 2:**
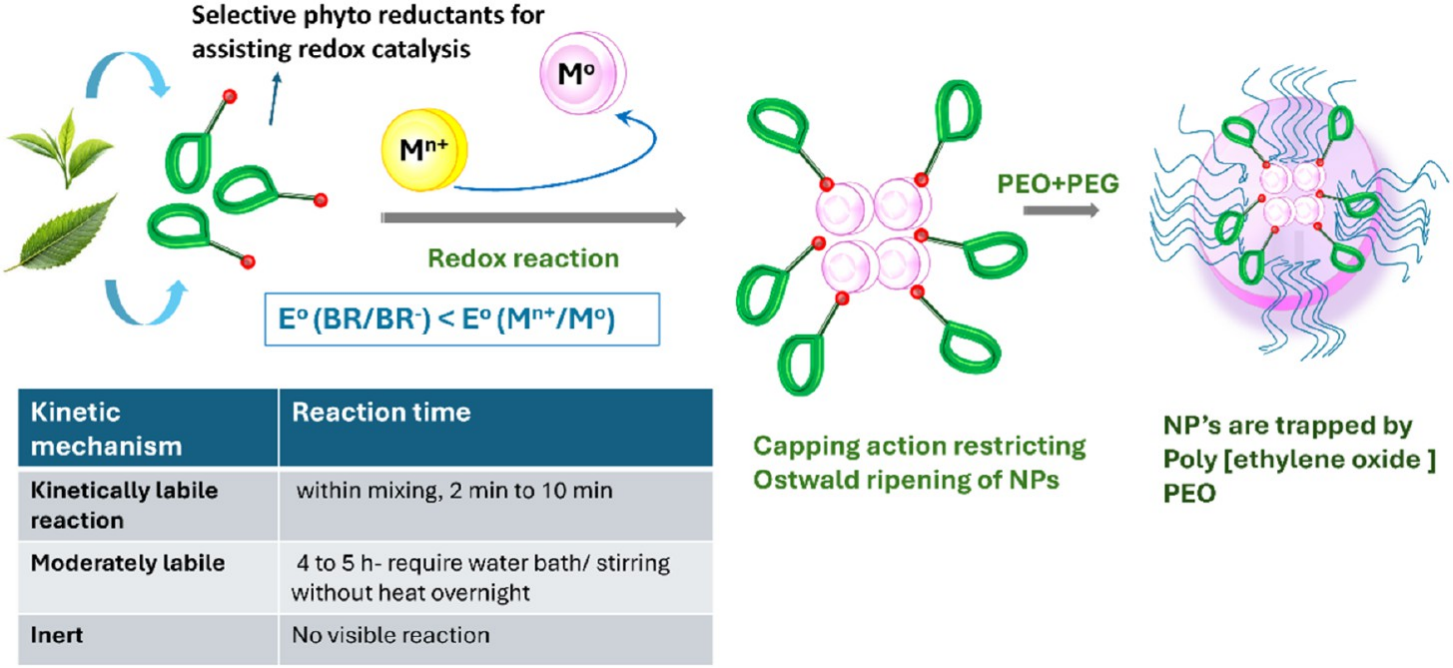
Schematic for the synthesis of metal nanoparticles using plant extract and trapping using a polymer (PEO) for the synthesis of SPE.

### 2.2 Synthesis of a Polymer Nanocomposite Film and Characterization

An appropriate amount of pristine poly­(ethylene oxide) [PEO] B.D.H, England, (molecular weight 10^5^) was allowed to mix using methanol as the solvent for 24 h. After 5–6 h, 0.1% poly­(ethylene glycol) (PEG) was added to the above mixture to retain the plasticizing action within the developed self-standing film. Different concentrations of Ag, Cu, and Fe NPs (nanoparticles), namely, 1, 3, 7, and 10 wt % were added to 20 mL of the already formed PEO solution (with PEG), which were then subjected to sonication (frequency of 20 kHz) for 1 h at room temperature.

This was followed by stirring for 3–4 h at room temperature until the solution was homogeneous. The solution mixture was allowed to deposit on glass substrates (maintaining uniformity) using the solution casting technique and was dried for 72 h in air, maintaining optimum relative humidity. The details of the samples are given in Table 1, and the schematic of the overall process in represented in Figure 2. The approximate thickness of the developed film was measured to be ∼200 μm. The films were studied for dielectric properties and ion conductivity using electrochemical impedance spectroscopy (Agilent 4294A-ecession Impedance Analyzer).

**Table 1:** Sample Identification of Plant Extract with Metal NPs and Polymer Nanocomposite Films^a^

plant extract	metal salt solution	reaction temperature (°C)	sample ID	sample ID of SPE with PEO as a host and 7 wt % metal NPs
*Camellia sinensis* (tea)	Ag^+^	25	Ag_T‑25_	PAg_T_
		40	Ag_T‑40_	
		90	Ag_T‑90_	
	Fe^2+^	25	Fe_T‑25_	PFe_T_
		40	Fe_T‑40_	
	Cu^2+^	25	Cu_T‑25_	PCu_T_
		40	Cu_T‑40_	
*Azadirachta indica* (neem)	Ag^+^	25	Ag_N‑25_	PAg_N_
		40	Ag_N‑40_	
		90	Ag_N‑90_	
	Fe^2+^	25	Fe_N‑25_	PFe_N_
		40	Fe_N‑40_	
	Cu^2+^	25	Cu_N‑25_	PCu_N_
		40	Cu_N‑40_	

a Note: 7 wt % loading of metal NPs has been selected as optimum as per the report mentioned in Section 3.

The EIS spectra were subjected to fitting using an equivalent circuit using Z-view software and are described in detail in the Supporting Information. The complex frequency responses (Z′ and Z″) were analyzed for the distribution of relaxation time (DRT) to correlate with frequency-dependent polarization processes. The resonating frequency was scanned in the range of 40–4 × 10^7^ Hz. The polarization behavior of the developed film was studied using the trend of EIS and the Bode plot. The phase purity of the developed PEO–metal NPs was studied using a Bruker-AXS diffractometer with Cu Kα radiation λ = 1.54 Å with a scan rate of 3° min^–1^. Thermal analysis of the PEO–metal NP composite was studied using differential scanning calorimetry (DSC) within a temperature range of 40–400 °C (Pyris Diamond DSC, PerkinElmer). The samples were scanned at a scan rate of 20 °C min^–1^. Microstructural characterization of the solid polymer film was studied using scanning electron microscopy (SEM FEIC-QUO-35357-0614 with a Bruker Quantax 100). The reported data on EIS, conductivity, and long-term endurance were reproduced using four sets considering different parts of the same polymer film and were recounted with error bars. This ensures the compositional homogeneity of the fabricated PEO films. The results for the PEO–metal NP composite films are reported with an NP loading of 7 wt %. This has been found to be optimum in terms of ion conductivity (shown in Figure 11) and hence only restored.

### 2.3 Theoretical Study on Bioactive Agents of *Camellia sinensis* and *Azadirachta indica*

Density functional theory (DFT) calculations of the chosen compounds were done by using the Gaussian 09 software package^36–38^ at the B3LYP functional^37^ combined with the 6-311G** basis set to obtain the optimized geometry and energy of the titled compounds. To express the normal modes in a molecular fixed coordinate system, a set of local symmetry coordinates were defined as recommended by Pulay et al.^39^ The Raman activities (*S*_i_) calculated by the Gaussian 09 program during the scaling procedure were converted to relative Raman intensities (*I*_i_) using the following relationship derived from the basic theory of Raman scattering.^40^

1 $$I_{i}=\frac{{f\left(v_{o}-v_{i}\right)}^{4}S_{i}}{v_{i}\left[1-\exp\left[\frac{-\mathrm{hc}v_{i}}{\mathrm{kT}}\right]\right]}$$

Here, ν_0_ is the excitation frequency (in cm^–1^), ν_i_ is the vibrational wavenumber of the ith normal mode, *h*, *c*, and *k* are the universal constants, and *f* is the suitably chosen common normalization factor for all of the peak intensities. The digital version of the observed and simulated spectra of the compound was used to identify the functional groups derived from the vibrational assignment.

DFT theory was very well used for the estimation of the vibrational spectra of the chosen compounds based on the optimized energy and potential energy distribution. The unscaled B3LYP/6-311G** vibrational frequencies are generally somewhat larger than the experimental value. However, for getting reliable information about the vibrational properties, the use of selective scaling is necessary. The calculated frequencies were scaled using a set of transferable scale factors as recommended by Pulay.^39,40^ The respective DFT study is represented and correlated with experimental findings through molecular orbital coordinates separated through the energy barrier required for transition from the ground state (HOMO, highest occupied molecular orbital) to available excited states (LUMO, lowest unoccupied molecular orbital). The study on thermos-energetics is undertaken using prior studies.^41–43^

## 3 Results and Discussion

### 3.1 Influence of Bioextract on Reduction for the Synthesis of Metal Nanoparticles Using UV–Vis Spectroscopy

The synthesis of Ag, Cu, and Fe nanoparticles (NPs) could be initially traced by qualitative means using the colorimetric change in the color of the solution, as depicted in Figure 1. The figure describes the saturation color of the solution after completion of the reaction, wherein the reaction time is dependent on the host–guest interaction of the bioreagent and the metal ion. Similar studies have been reported by many researchers to mark the initialization of NP synthesis through the bioreduction route.^10,11,13,14^ The bioreagent(s) within herbs *Camellia sinensis* and *Azadirachta indica*, as tabulated in Table S2, function as reducing agents as per the comparative trend of reduction potential with respect to redox couples such as Ag^+^|Ag^0^ (0.34 V), Cu^2+^|Cu^0^ (0.34 V), and Fe^2+^| Fe^0^ (−0.44 V).^44^ As per the magnitude, it could be stated that Cu^2+^ bears a greater tendency to be reduced compared to Ag^+^ and is kinetically more active as per the standard reduction potential. In contrast, Fe^2+^ suffers easy oxidation and is reluctant to be reduced, which is also supported by the negative reduction potential of the redox couple. In this aspect, green synthesis plays a significant role in reducing Fe^2+^ through a subsequent charge transfer process (conjugated electron cloud within the bioreagent), as explained in Section 3.2. Furthermore, the herbs consist of multiple bioreagents (phytochemicals; listed in Table S2 along with the reduction potential/mode of interaction with the cation), which also act as capping agents.^30–35^ As shown in Figure 2, the phytochemicals present within the herbs also function as effective capping agents and restrict the ripening of nanoparticles after nucleation (Figure 7) and thereby stabilizing them.

The initial technique used for the characterization is UV–vis spectroscopy, since the formed NPs in general respond at a wavelength within 200–400 nm.^45,46^ During the formation of metal NPs, the resulting surface plasmon comprises the wavering collection of collective conduction charges (electrons), which are perturbed by the source electromagnetic spectrum.^46^ In the case of nanoparticles synthesized through a chemical process, the excitation band limit depends on the electronic density of state, size, and shape of the NPs produced.^9^ UV–vis spectra for the formation of Ag, Cu, and Fe NPs are shown in Figures 3–5, respectively. The absorption spectra of the reaction mixture of Ag^+^ with tea and neem extract consist of plasmon bands of Ag^0^ centered at 443 and 432 nm at 25 °C.

**Figure 3:**
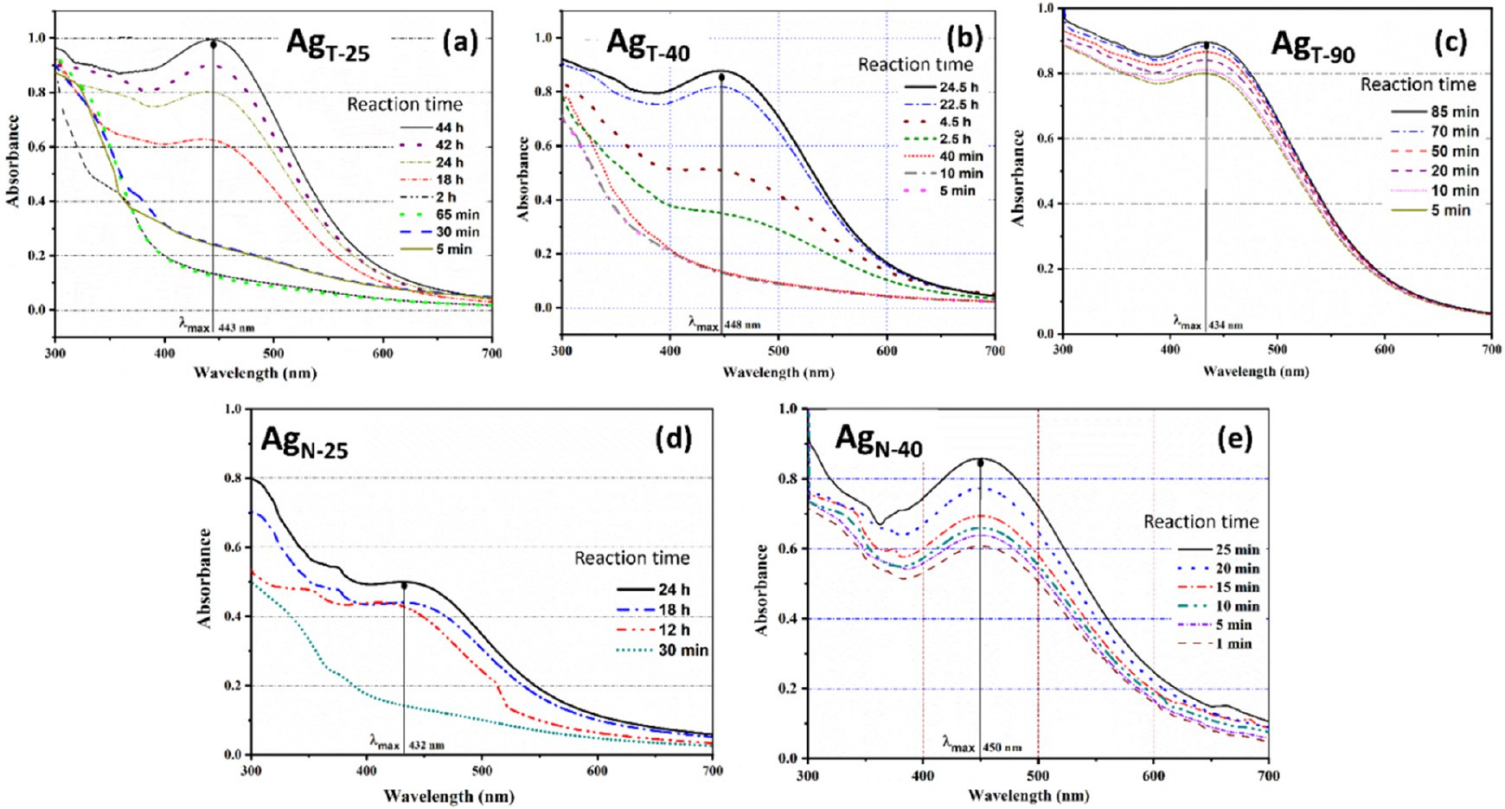
UV–vis spectra for Ag NPs synthesized using the extract of *Camellia sinensis* (a–c) and *Azadirachta indica* (d, e) as a function of reaction temperature: 25 °C (a, d), 40 °C (b, e), and 90 °C (c).

**4:**
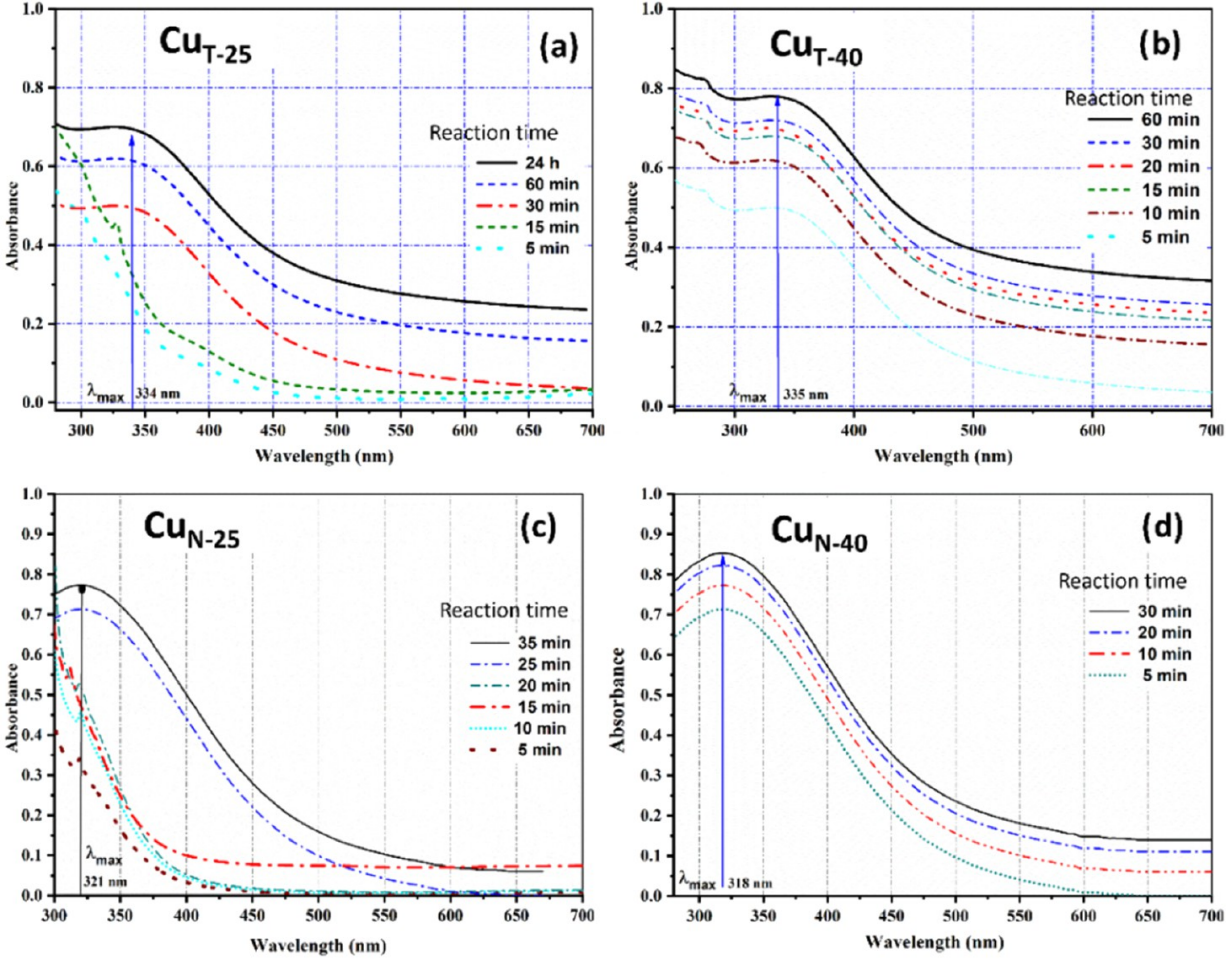
UV–vis spectra for Cu NPs synthesized using the extract of *Camellia sinensis* (a, b) and *Azadirachta indica* (c, d) as a function of reaction temperature: 25 °C (a, c) and 40 °C (b, d).

**Figure 5:**
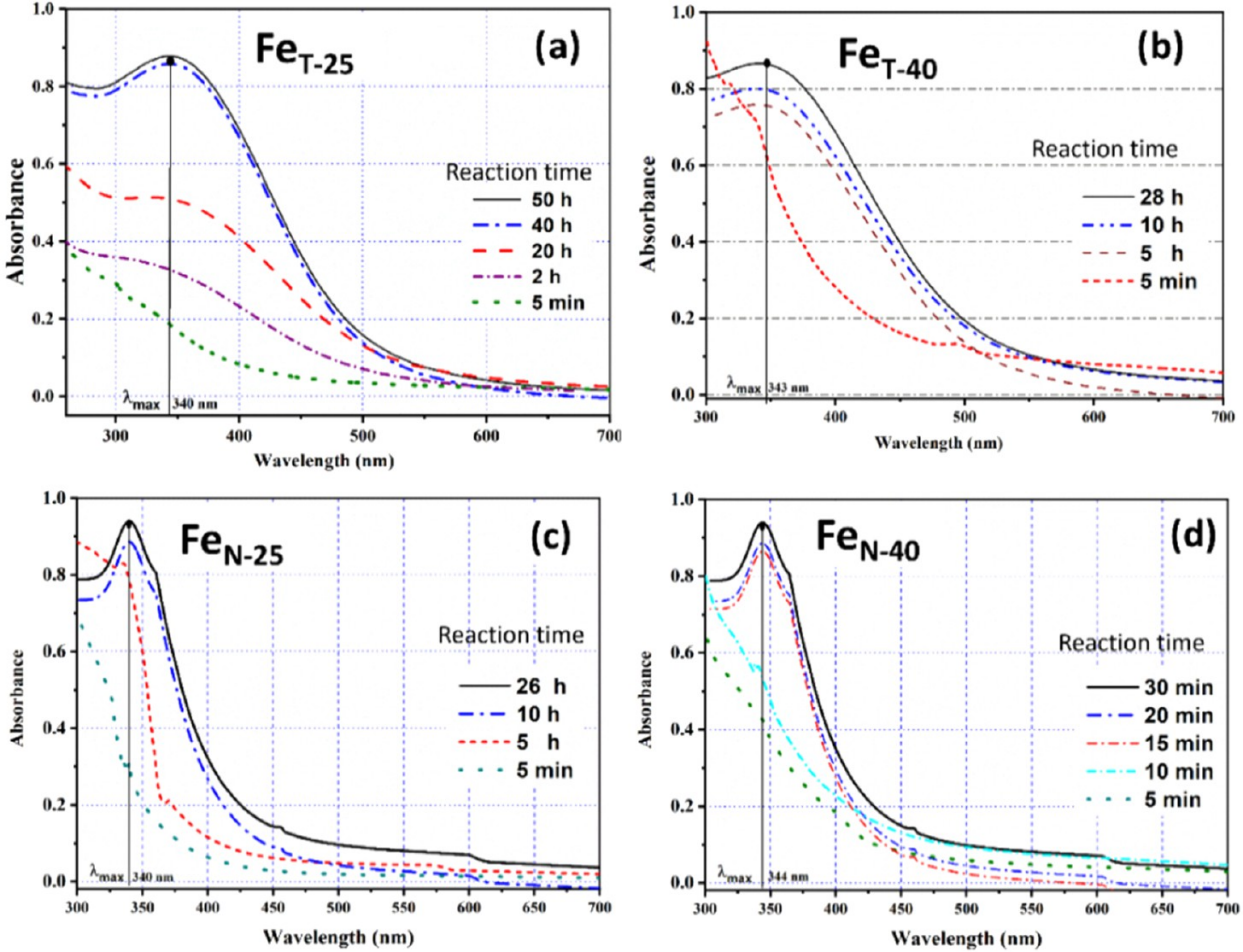
UV–vis spectra for Fe NPs synthesized using the extract of *Camellia sinensis* (a, b) and *Azadirachta indica* (c, d) as a function of reaction temperature: 25 °C (a, c) and 40 °C (b, d).

However, with an increase in reaction temperature (until 90 °C for tea and 40 °C for neem extract), the maxima pertaining to maximum absorption bands slightly change based on thermal activation. It could be noted that neem extract tends to accelerate the redox reaction and requires much less time for completion of the reaction, irrespective of the type of metal salt (Ag^+^, Fe^2+^, and Cu^2+^) compared to the phytochemicals present in tea. The colloidal solution of Ag^+^ requires 48 h for complete reduction using bioreagents of tea, whereas 24 h for the same upon employing neem. Owing to the sluggish rate of reaction for the Ag_T_ system, the reaction temperature was increased to 90 °C. Ag_T‑90_ does not exhibit any initialization/propagation phase of the reaction, as can be seen from Figure 3c, wherein, with 5 min of reaction, it proceeds toward completion (labile reaction).

The high temperature for Ag_T_, therefore, is found to be progressive toward nucleation and growth of NPs. However, a further increase in the reaction temperature is not recommended, as it results in decomposition of precursors before they can reduce and form nucleating centers. In addition, over nucleation results in ripening of formed NPs (due to the presence of high surface energy) and forms coagulation.^47^ The Ag_N_ system, however, requires an initial 24 h to saturate the reaction at 25 °C, which accelerates within 25 min when undertaken at 40 °C. The faster redox reaction also tends to shift the plasmon bands of Ag^o^ centered at 450 nm from 432 nm (at 25 °C). Compared to the Ag+/Ag^0^ system, the Cu^2+^/Cu^o^ transition requires much less time (24 h with tea and 35 min with neem extract) @25 °C and (60 min with tea and 30 min with neem extract) @40 °C. Upon increasing the reaction temperature, the plasmon bands of Cu^o^ change negligibly for the Cu_T_ system (334/335 nm) and from 321 to 318 nm for Cu_N_.

The lability of Cu^2+^/Cu^o^ (as indicated from a lower standard reduction potential, 0.34 V) is further corroborated upon interaction with reactive phytochemicals from neem, which does not show any significant temperature dependence for the completion of the reaction (∼30 min).

The significant outcome of green synthesis is noted from the formation of Fe NPs, since the redox couple (Fe^2+^/Fe^o^) shows a spontaneous tendency to get oxidized. Tea results in a higher reduction tenure of ∼50 h @ 25 °C, which reduces to 28 h upon promoting thermal-aided chemical reaction@40 °C. As is evident, the resonating wavelength corresponding to the formation of Fe^o^ suffers a slight red shift with an increase in reaction temperature from 25 to 40 °C. The reported bands stay persistent upon standing the reaction mixture for 24 h after completion of the reaction, which signifies reasonable stabilization of the experimental dispersed solution.

### 3.2 Characterization of Metal NPs

The characteristics of metal NPs formed from variable biosources may vary sometimes, as per the report of prior studies.^10–16^ This is possibly due to the compositional alteration of phytochemicals present in the bioextract. The variability in reducing agents could affect the pattern of size distribution, shape, morphology, etc. Microstructural analysis using SEM reveals the formation of spherical nanoparticles (Figure 6) for Ag, Cu, and Fe. The Fe NP (Figure 6c) exhibits agglomerated particles with a broad particle size distribution, averaging 32.5 nm in diameter. In contrast, the Cu and Ag samples display relatively smaller average particle sizes. The Cu sample has a significantly narrower size distribution with an average particle size of 23 nm, whereas the Ag sample shows a wider size distribution, averaging 5.7 nm. Notably, unlike the Fe sample, the Cu and Ag samples do not exhibit particle agglomeration.

**Figure 6:**
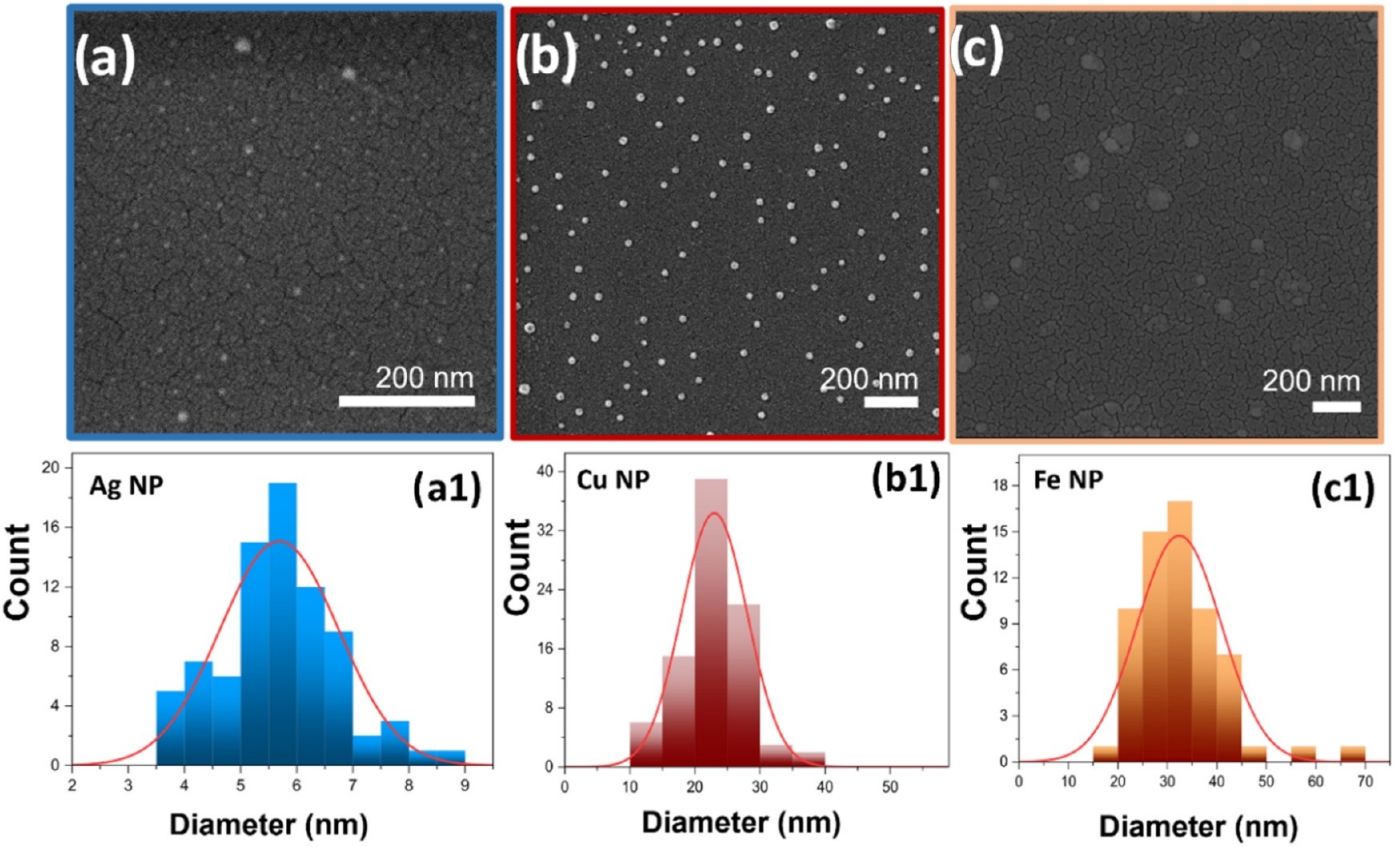
SEM micrograph of metal nanoparticles of (a) Ag, (b) Cu, and (c) Fe along with their particle size histograms (a1, b1, and c1).

The histogram corresponding to the particle size distribution is found to be unimodal, which suggests negligible ripening during stage 2 (Figure 8). This further establishes the effective capping action of the bioreagents (phytochemicals) present within the plant extract. Variation in biosources (neem and tea here) affects the nature of NPs produced. The particle size distribution (PSD) reported in Figure 6 is the mean of five sets of SEM measurements that do not undergo any abrupt variations

### 3.3 Mechanistic Overview and Theoretical Study on Green Synthesis of Metal NPs

The theoretical study reported herewith elucidates the energetics of selected phytochemicals, which could potentially act as the bioreductant for Ag^+^/Cu^2+^/Fe^2+^. As per the study of prior arts, bioreduction of metal NPs involves three primary stages, namely, (a) activation stage; (b) growth (or propagation stage); and (c) termination stage. The initial activation process^48^ requires Gibbs’ free energy (ΔG) of the redox couple to be spontaneous as per eq 2

2 $$\Delta G=-\mathrm{nF}E_{\mathrm{cell}}^{o}$$

where *n*, F, and *E*_cell_^o^ represent the number of electrons transferred, Faraday’s constant, and cell potential of the redox couple.

Furthermore, for green synthesis, *E*_cell_^o^ could be represented as

3 $$E_{cell}^{o}=\left|\left(E_{BR/BR\left(-\right)+H\left(+\right)+n*elect\mathrm{ro}n}^{o}\right)-\left(E_{M\left(n+\right)/M\left(o\right)}^{o}\right)\right|$$

where BR represents the bioreagent.

The selection of BR (bioreagent) within the herb acting as the reducing agent depends on the comparative reduction potential with respect to the (M^*n*+^/M^o^) redox couple, so that the net activation barrier remains to be spontaneous (marked by negative Δ*G*). Hence, despite using a similar herb as the phytoreductant source, the selectivity of the active reducing agent is dependent on the metal NP being produced as the active product.

The formed metal NPs from the first stage being in atomic form are highly unstable owing to the presence of higher surface energy and thereby tend to undergo spontaneous nucleation in stage 2 (Figure 7), also termed as Oswald ripening. The shape of the formed NPs varies as per the host–guest interaction with the associated capping agents present in the herb and the thermoenergetics governing the kinetic process (Figure 2). Finally, the nucleating metal NP is stabilized by the capping agents present in the herb (Figure 7). The mode of the redox process (primarily oxidation) underlying the active phytoreductant involves tautomeric transformation, wherein keto–enol transformation is obtained, involving active hydrogen linked to the flavonoid(s) (Figure 7).^49^ The functional groups that assist the plants in acting as reducing/stabilizing agents are primarily hydroxyl and carbonyl groups (Table S1).^50^ Reports suggest that termination could involve either resonance stabilization of the active phytoreductant (superposition of pi-electron) or complexation of polyphenols (or flavonoids) with formed metal NPs.

**Figure 7:**
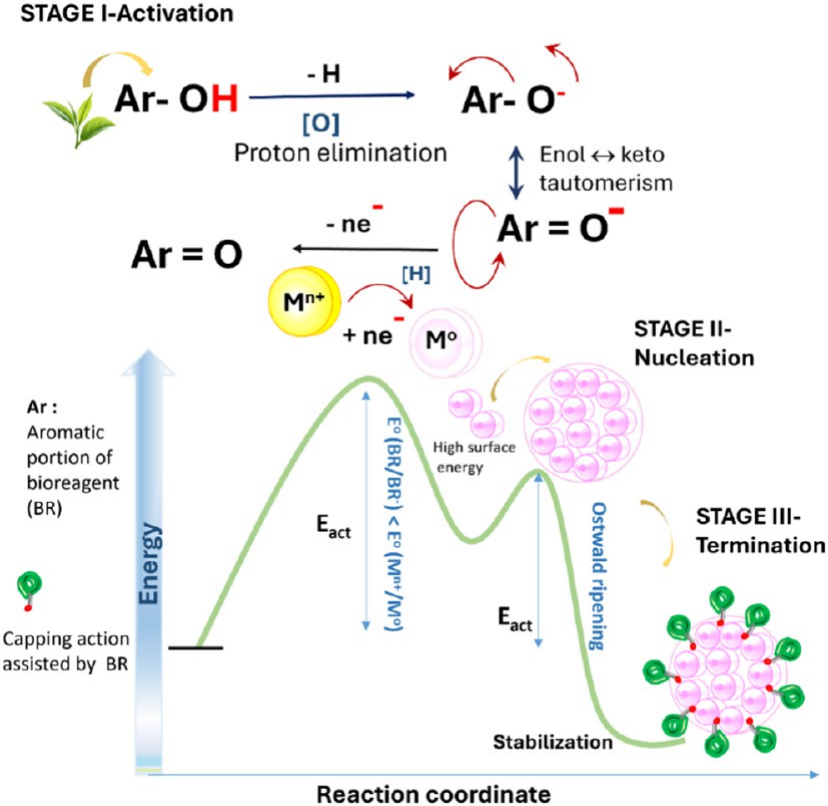
Schematic of chemical mechanism for the synthesis of metal NPs using the green synthesis route.

The authors have tried to analyze the possible contribution of a bioreagent that could act as a potent reducing agent in the present study. Theoretical analysis has been undertaken using density functional theory (DFT) to optimize selected phytochemicals of neem and tea. The respective molecular orbitals (HOMO and LUMO) are represented in Figures S1–S4. For quantitative analysis, the energy level (band) diagrams for HOMO and LUMO as a function of available phytochemicals are shown in Figure 8a,b.

**Figure 8:**
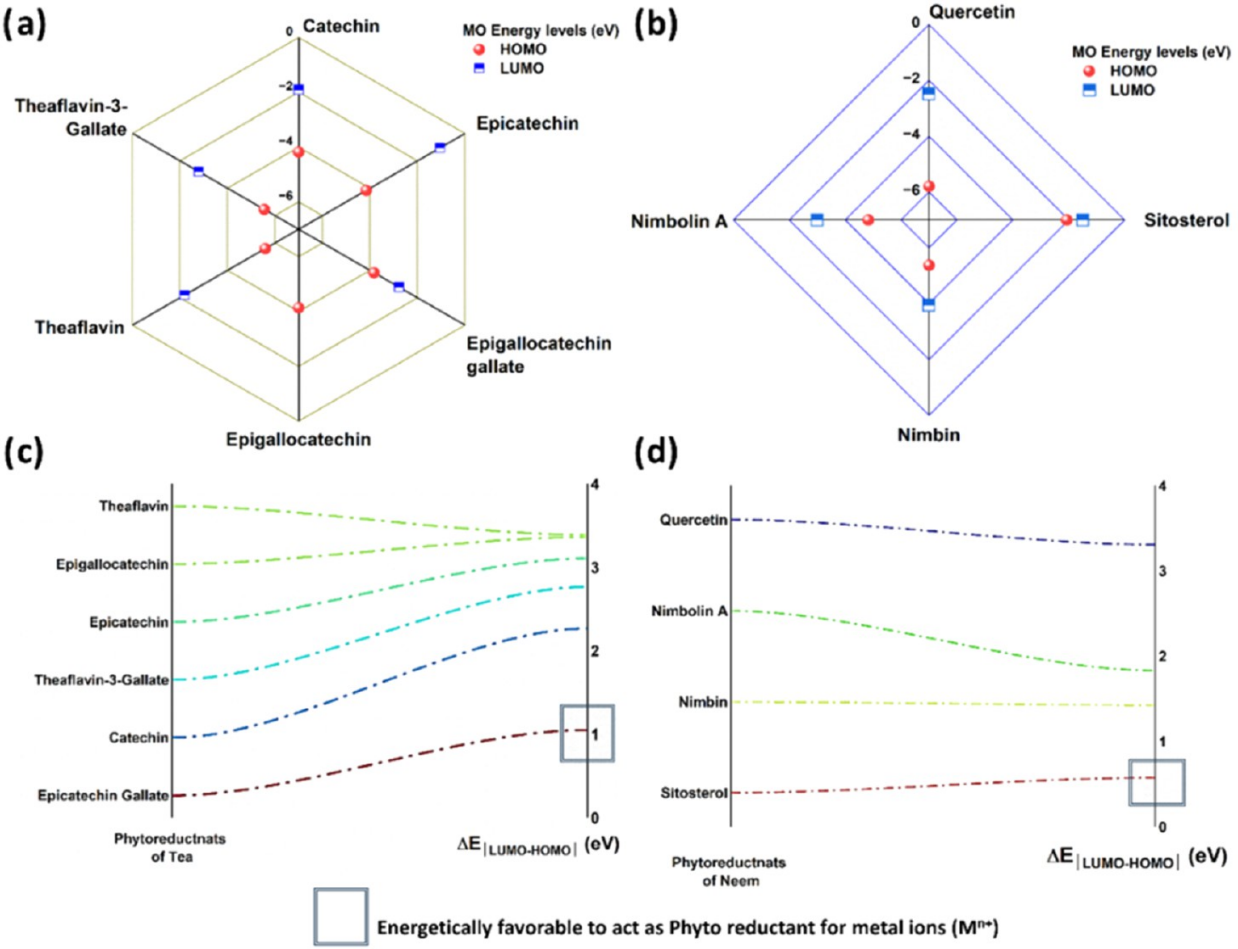
Molecular orbital energy levels for the phytoreductants available in (a) tea and (b) neem. Representation of Δ*E*_MO energy gap_ evaluated from DFT as a function of phytoreductants available in (c) tea and (d) neem.

Hence, in addition to the intrinsic standard reduction potential necessities for reducing M^*n*+^, the transition energy gap, as indicated in Figure 8, is more significant to control the reactivity as reductant species for experimental cations (Ag^+^, Cu^2+^, and Fe^2+^). The bioreagent with the least band gap is reactive and could act as a potent phytoreductant for initiating the redox reaction, followed by subsequent capping. It could be noted that the transition energy (band gap, Δ*E*_|LUMO–HOMO|_) is minimum for Epicatechin gallate (tea, 1.0531 eV) and Sitosterol (neem, 0.5744 eV), as shown in Figure 8c,d. Hence, the mentioned compounds could act as potential reducing agents. The final stage of termination is, however, controlled by the secondary bonding capability of the available bioreagents for acting as capping reagents.

### 3.4 Characterization of M^0^@PEO Composite Films

#### 3.4.1 X-ray Diffraction and Thermal Analyses of the M^0^@PEO Composite Film

The phase purity of the PEO–metal NP films is studied using X-ray diffraction, as shown in Figure 9. In all of the self-standing films, the primary diffraction peaks of PEO are visible at 19.5 and 23.6°, which correspond to the (120) and (112) planes, respectively.^51^ Figure 9 shows the XRD spectra with metal NP loadings of 7 and 10 wt % to elucidate the influence on the % crystallinity of the PEO film. The degree of crystallinity^52^ is calculated by the following equation

4 $$\%\;\mathrm{crystallinity}=\left({\mathrm{area}}_{\mathrm{crystalline}}/\mathrm{total}\;\mathrm{area}\right)\times 100$$

**Figure 9:**
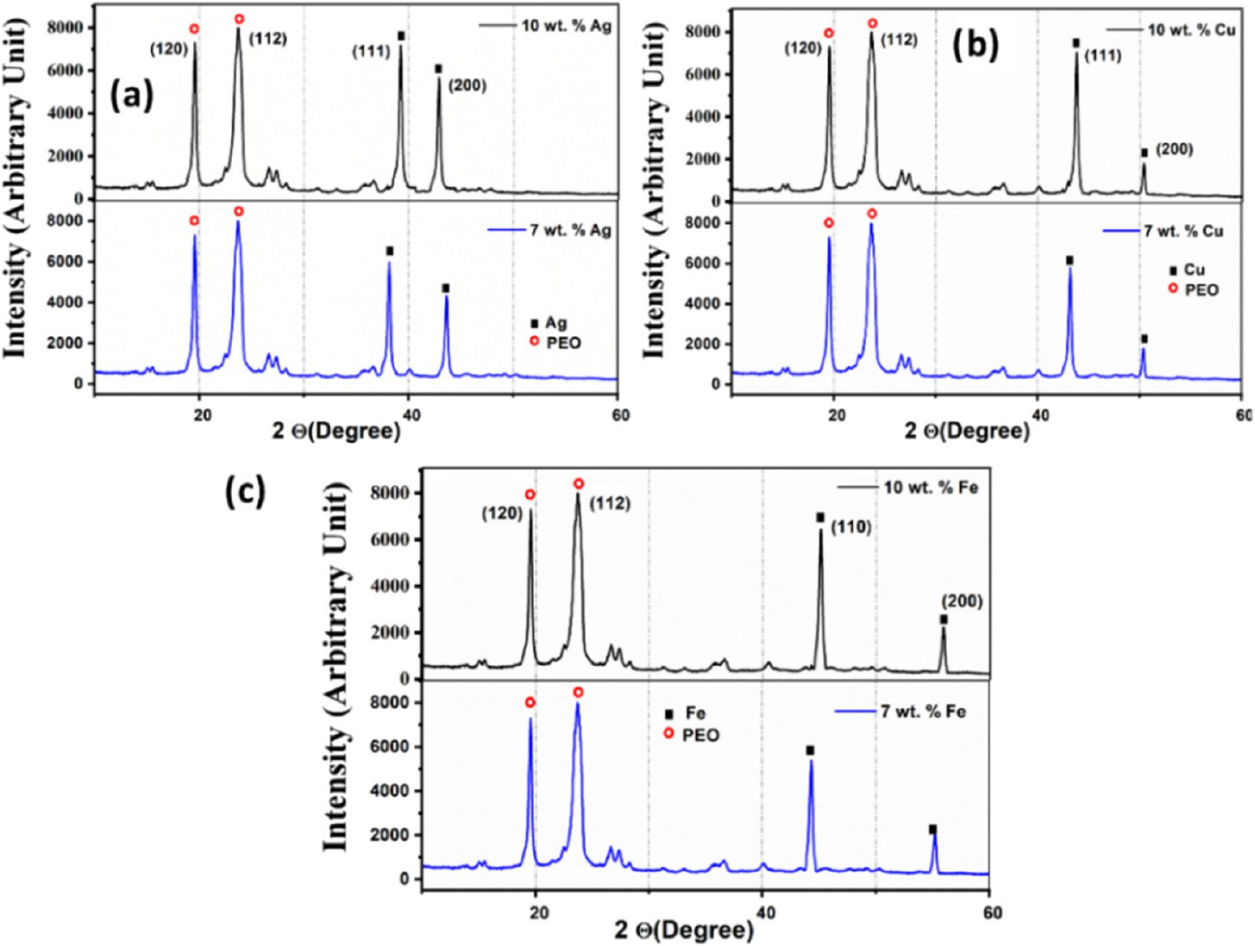
X-ray diffraction study of (a) P_Ag_, (b) P_Cu_, and (c) P_Fe_ having nanoparticle dopings of 7 and 10 wt %.

Patla et al.^21,22^ earlier demonstrated the % crystallinity of pristine PEO to be ∼24% (w.r.t 78% obtained using DSC). This accounts for the arbitrary ratio of intensities considered during XRD analysis, whereas DSC accounts for the variation in enthalpy during melting phenomena being more accurate. However, a comparative analysis could be studied that shows a significant reduction in the % crystallinity of the PEO matrix upon incorporation of metal NPs. Upon changing the loading of NPs from 1 to 10 wt %, crystallinity is found to reduce until 7 wt % followed by a steep increase at 10 wt %. At 7 wt % NP loading, 19, 22, and 23% magnitudes of % crystallinity are observed for P_Ag_, P_Cu_, and P_Fe_, which significantly increase to 34, 32, and 31% at 10 wt % NP loading. With reference to the prior studies, it could be stated that the insertion of metal NPs tends to restrict the crystallization of the host polymer and enhance the amorphous matrix. A plausible explanation could be an interaction of polar groups of polymers with the surface functionalities of NPs (secondary bonding), which generates in situ formation of certain complexes, thereby limiting the growth of long-range ordering of crystalline phases. This is also correlated with an increase in the segmental motion of the matrix at optimum doping of metal NPs.^53^

On the contrary, with a subsequent increase in the doping of metal NPs (10 wt % in the present context), self-association or aggregation occurs within the nanoparticles (owing to the intrinsic higher surface energy). This increases the probability of active hydrogen bonding among formed nanoaggregates and the host polymer, thereby ordering the overall matrix to form spherulites.^54,55^ Hence, the optimum doping of metal NPs has been selected to be 7 wt % in the present research, and the results in the subsequent sections are only reported for the said composition. The increase in the amorphous region until 7 wt % doping with NPs could also be corroborated with enhanced ionic conductivity of the polymer composite film in Section 3.4.2. The XRD spectra reported in Figure 9 for PEO–metal NPs correspond to the use of both experimental herbs neem and tea as bioreagents.

The XRD peaks corresponding to 38.1 and 44.6° (Figure 9a) account for the formation of Ag NPs for (111) and (200) Bragg’s planes, respectively (JCPDS card no. 65-2871).^56^ Similarly, FCC, Cu NPs are ascertained by the peaks at 43.4 and 50.5° for (111) and (200) planes, respectively (JCPDS card no. 04-0836).^57^ The XRD peaks are indexed for phase-pure Fe NPs at 44.28 and 55.2° for (110) and (200) planes, respectively (JCPDS code of 01-075-1550).^58,59^ All of the synthesized PEO composite films are therefore reported to be phase pure without having any impurity phase.

The results of thermal analysis are given in the form of differential scanning thermograms (DSC) in Figure 10. Alqadi et al.^60^ studied that the pristine PEO film exhibited a sole endothermic peak at ∼76 °C, corresponding to the melting of the polymer. On a similar note, Patla et al. also reported a single endothermic peak of PEO having a small ratio of inorganic salts.^23^ However, upon insertion of metal NPs, an additional exothermic peak is noted in all of the spectra, as shown in Figure 10. This is cited as the crystallization temperature, which is also influenced by the type of phytoreductant used and the loading of metal NPs. The details of melting and crystallization temperatures as a function of the synthesized PEO film are tabulated in Table 2.

**Figure 10:**
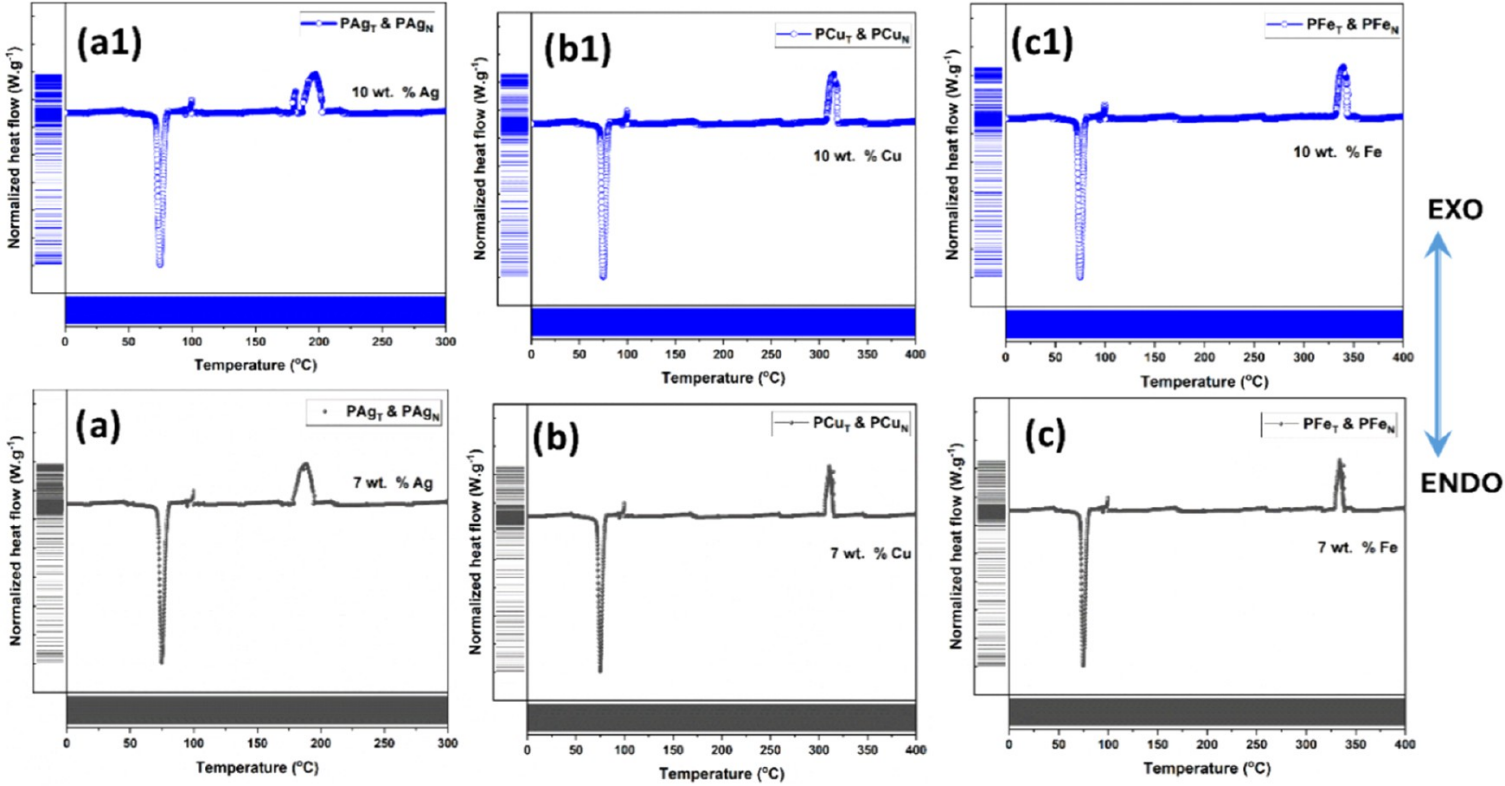
Differential scanning thermogram of (a, a1) P_Ag_, (b, b1) P_Cu_, and (c, c1) P_Fe_ having nanoparticle dopings of 7 wt % (a, b, c) and 10 wt % (a1, b1, c1).

**Table 2:** Variation of Melting and Crystallization Temperature and % Crystallinity as a Function of Synthesized PEO Films

sample ID	loading of metal NP (wt %)	melting temperature (*T*_M_) (°C)	crystallization temperature (*T*_C_) (°C)	% crystallinity
PAg_T_ & PAg_N_	7	75	188.5	70.2
	10	75.2	196	83.4
PCu_T_ & PCu_N_	7	75.05	310.5	74
	10	75.5	315.5	81.2
PFe_T_ & PFe_N_	7	75.05	333.9	76
	10	75.06	338.7	79.6

Irrespective of the type of NP, the crystallization temperature (*T*_c_) is found to be slightly increased with an increase in the loading of NPs from 7 to 10 wt %. Cobos et al.^61^ suggested that the enhanced nucleating influence of metal NPs is responsible for the increase of *T*_c_, as tabulated. In addition, there are reports that support the lowering of the melting temperature (*T*_m_) upon insertion of metal NPs.^62^ This is, however, established in the present study, wherein the insertion of metal NPs lowers the melting temperature of the PEO composite. % crystallization is also calculated from DSC using the following equation

5 $$\%\;crystallinity=\left(\frac{{\Delta H}_{m}-{\Delta H}_{c}}{{\Delta H}_{m,0}}\right)\times 100$$

where Δ*H*_c_, Δ*H*_m_, and Δ*H*_m,0_ are the enthalpy of crystallization and melting and theoretical melting enthalpy of PEO considering 100% crystallinity, respectively. Δ*H*_m,0_ has been taken as 203 J g^–1^ as per the report of Alsaad et al.^63^

The magnitude of crystallinity calculated using enthalpy is higher compared to that obtained using XRD, as already mentioned. However, the trend of crystallinity with variation in metal NP loading is similar to that predicted using XRD analysis. At the optimum loading of metal NPs of ∼7 wt %, the amorphous region within PEO increases and reduces the long-range order in the composite. However, with a subsequent increase in the degree of NP loading, crystallinity increases significantly. This is directly related to the ion conduction path within the dual phase matrix and is discussed in Section 3.4.2. Apart from some smaller artifacts, the DSC pattern of PEO–metal NP composite is independent of the source of phytoreductant utilized.

#### 3.4.2 Ion Conduction in M^0^@PEO Composite Films

The DC conductivities for the PEO–metal NP films shown in Figure 11a,b are extracted from the EIS, wherein the complex phase lag is absent. It could be noted from Figure 11a that irrespective of the type of metal NP, the ionic conductivity of the PEO film is found to be increased approximately by two orders upon increasing the loading of nanoparticles from 1 to 7 wt %. With an increase in the % of loading, the charge density of mobile ions increases; however, the mobility is found to be influenced by the resultant ion pairing^64^ at significantly higher doping of metal NPs.

**Figure 11:**
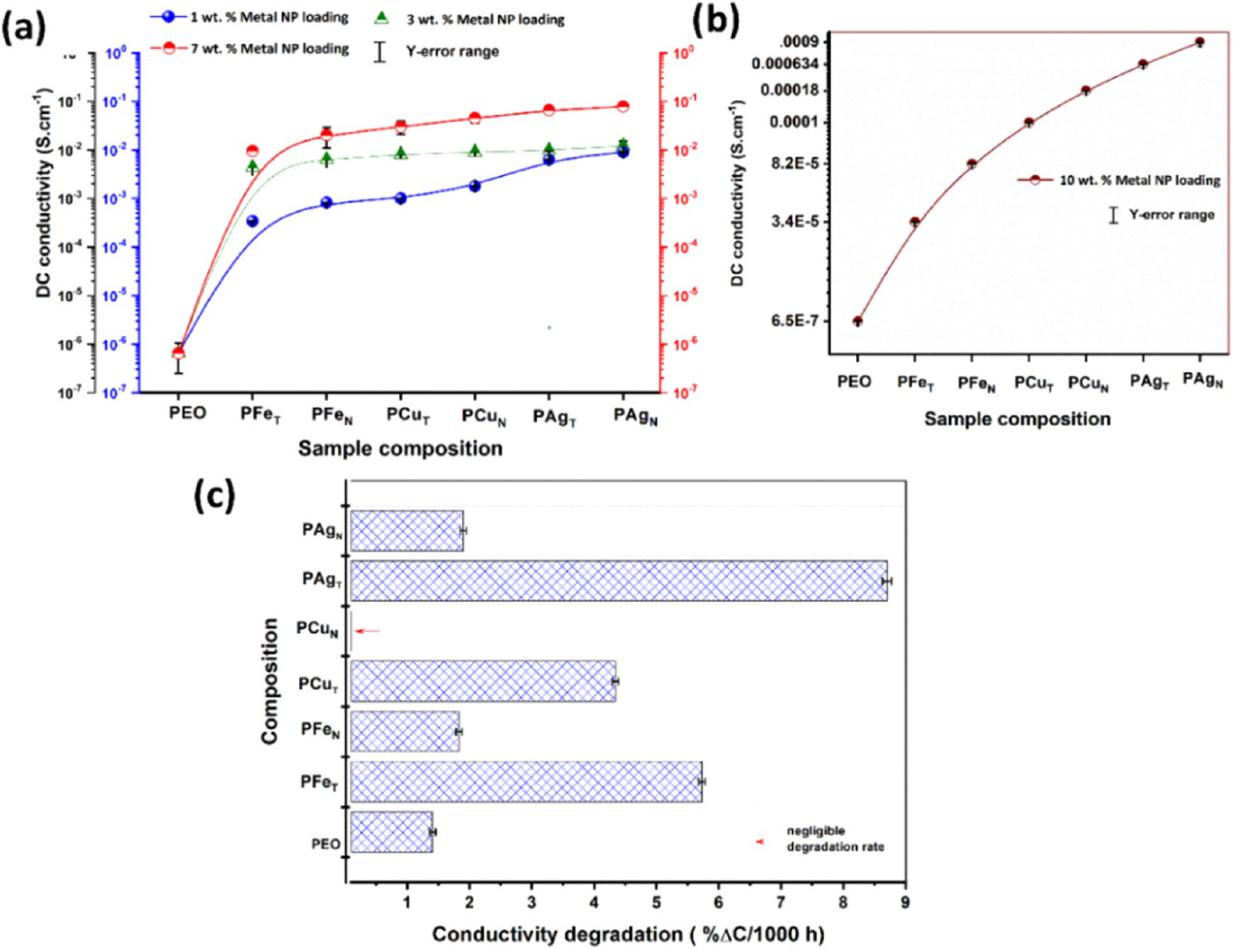
(a) Trend of DC conductivity as a function of PEO–metal NP composite films with respect to NP doping of 1–7 and 10 wt %. (b, c) Endurance study of the PEO composite film in terms of conductivity degradation studied for 2000 h (negligible conductivity degradation is marked in a red arrow for better visibility). * Error analysis is marked by bars at relevant axes.

However, at 10 wt % doping (Figure 11b), the movement of ions is restricted, which shows a sharp reduction in the magnitude of ionic conduction. This could be correlated with the formation of an ion pair at higher loading of metal NPs. In addition, microstructural evaluation shows noteworthy ripening of the metal particles with the lamellar growth of PEO as well (Figure 13a–e) at 10 wt % doping. Hence, 7 wt % doping is selected as the optimum concentration required for the property improvement of PEO films. The SEM images of PEO–metal NPs with 7 wt % loading are shown in Figure 12a–f. It is mentioned in Section 3.2that Fe NPs have a higher tendency to get agglomerated (Figure 6c,c1). A similar ripening tendency is visible in Figure 12c,f, compared to the PEO matrix with Ag and Cu NPs (Figure 12a,b,d,e). The morphological difference among PEO matrices having the same NPs [Figure 12a–f] could be accounted for by the influence of phytochemicals (from neem and tea). The more dispersed Ag NPs within the host PEO matrix result in the highest ion conduction (in all doping concentrations) owing to excellent hopping with a maximum mean free path. It could also be visible from the SEM and corroborated by DSC and XRD that the spherulite distribution is less in the PAg system.

**Figure 12:**
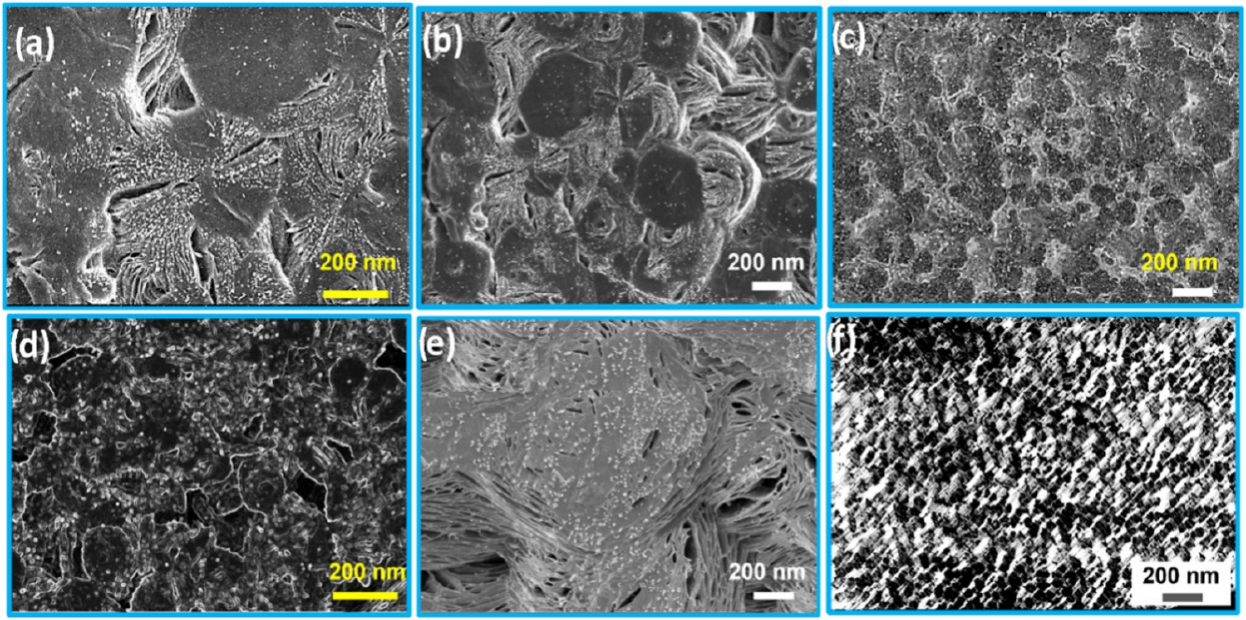
SEM images for (a) PAg_N_, (b) PCu_N_, (c) PFe_N_, (d) PAg_T_, (e) PCu_T_, and (f) PFe_T_ with 7 wt % loading of NPs.

Due to higher amorphous regions within such a matrix, the transporting ion suffers from a minimum tortuous path and accounts for maximum ion conductivity. Consequently, the ion conduction in the PCu system is lower compared to PAg due to increased crystallite regions (74% compared to 70% in the PAg system).

As expected, with higher % crystallinity (76%) with the ripening influence of Fe NPs, the film shows minimum ion conduction. As discussed in Section 3.4.1, % crystallinity increases abruptly with higher doping of 10 wt % due to an increase in crystalline sites (Figure 13a–f). This results in a multifold reduction of ion conduction in PEO–metal NP films with 10 wt % loading (Figure 11b). In addition, at such higher doping % of NPs, ion pairing is obtained,^64^ which reduces the net ionic conduction within PEO, due to the decrease in the available mean free path, which increases the tortuosity within the polymer matrix. Mukhopadhyay et al.^65^ reported the influence of tortuosity for optimizing the morphology of metal ceramic composites.

**Figure 13:**
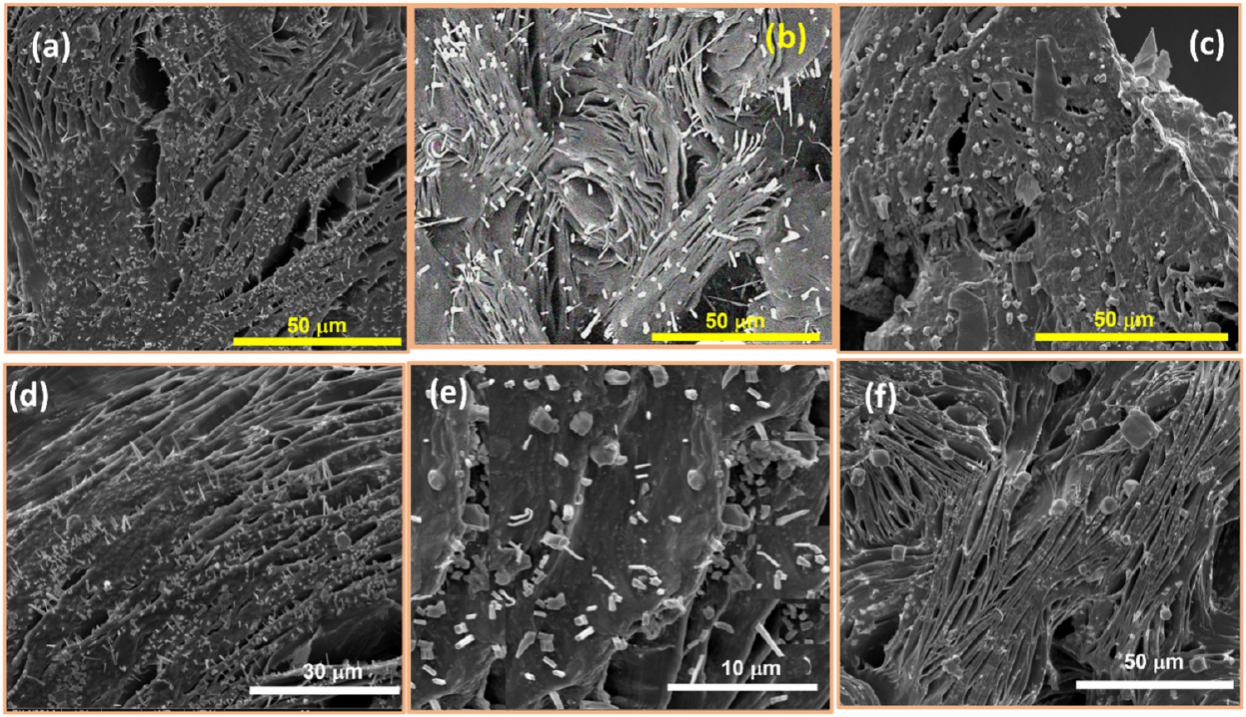
SEM images for (a) PAg_N_, (b) PCu_N_, (c) PFe_N_, (d) PAg_T_, (e) PCu_T_, and (f) PFe_T_ with 10 wt % loading of NPs.

An excellent correlation among the morphology of PEO–NP composite film(s) with a degradation study on ionic conductivity for 2000 h is reported in Figure 11c. Relatively higher deterioration of PAg, PFe, and PCu systems is observed compared to pristine PEO. This could be accounted for based on the higher tendency of nanoparticles to coarsen despite being capped compared to the pristine host polymer. Compared to tea, the bioreagents of neem are found to be more effective toward capping the nanoparticles, thereby exhibiting a lower rate of degradation in ionic conductivity. However, as per the tendency of agglomeration, Fe NPs (Figures 6c,c1 and 12c,f) result in significant deterioration for PFe_T_ (5.72%/1000 h) compared to PFe_N_ (1.8%/1000 h). For Ag NPs, which are smaller (finer, Figure 6a,a1), the agglomeration is still higher during on-load conductivity measurement. This results in maximum deterioration of 8.7%/1000 h for PAg_T_. Neem acts as a better capping agent; the conductivity degradation of PAg_N_ is comparatively lower (1.9%/1000 h). In contrast to Ag and Fe NPs, Cu NPs suffer from the least degradation. The redox kinetics of Cu^2+^/Cu^0^ is labile, which results in optimum distribution of particle size for Cu NPs. Tested for 2000 h, PCu_N_ does not exhibit any degradation, as reported in Figure 11c. Therefore, for long-term application of polymer films, the composite with Cu NPs is suggested for future study.

Polymer composites are complex systems comprising amorphous and crystalline regions and are termed macromolecules. Due to their segmental motion (localized) and molecular rotation and translation (global), the mode of relaxation is complex when perturbed with any external frequency (or heat) source.^23–26^ The complex relaxation consists of a linear resistive part (represented by DC) and nonlinear polarizations (marked by AC in Figure 14, having a positive phase lag). The ion conduction corresponding to nonlinear polarization is shown in Figure 14, wherein the frequency dependence is clearly visible. The nonlinear dependence is marked by charge transfer (or ion movement) and diffusional transit of involved ions. Compared to ions imparted by inorganic salts within the PEO matrix, as studied in previous reports,^23,26^ ion conduction in PEO–metal NP films is more facile, which is explained based on the ion hopping mechanism. The disperse trait of ion conduction [σ­(f)] at higher frequency (*f* = 10^6^–10^7^ Hz, Figure 14a) can be correlated with the mean square displacement (*d* ^2^) of mobile ions,^66^ as shown in eq 6

$$\sigma \left(f\right)=-\left(Nq^{2}f^{2}/6\mathrm{kT}\right)\int_{0}^{\infty }\langle d^{2}\rangle e^{-\mathrm{ift}}\mathrm{dt}$$

where *q*, *k*, *T*, and *t* correspond to the charge, Boltzmann’s constant, applied temperature, and time, respectively. It can be observed that ion conduction is frequency-independent within a lower range, whereas it starts responding with the field at higher applied frequency. Similar to Figure 11a,b, the trend in AC conduction is the highest conductivity for PAg_N_ and the lowest for the PFe system. At higher applied frequency (10^6^–10^7^ Hz), ion conduction is observed to be frequency-dependent, wherein the ion hopping mechanism is operative. Hence, the mean square displacement of the ion is smaller at such higher frequencies.

**Figure 14:**
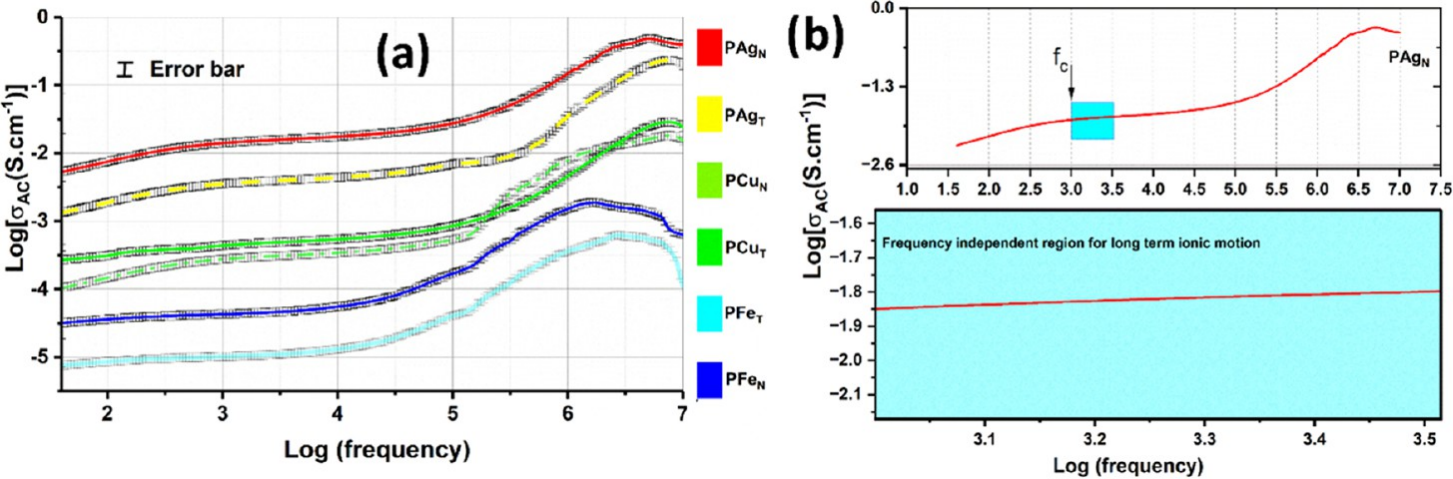
(a) Frequency-dependent AC conductivity as a function of PEO–metal NP composite films with respect to an NP doping of 7 wt % with error bars and (b) zoomed plot for PAg_N_ to elucidate the crossover frequency region (*f*_c_) for the frequency-independent region.

However, long-range ionic conduction is observed at relatively lower frequency, wherein the dependence is neutral. This region is marked by crossover over frequency (*f*_c_), which favors the unrestricted random walk of the associated ions (Figure 14b, represented for only PAg_N_ as an example). The AC conduction trend for PEO–metal NPs with lower loadings of 1–3 wt % and the highest loading of 10 wt % is shown in Figure S5. This clearly signifies the role of higher crystalline regions formed with 10 wt % NPs in reducing the ion conduction mechanism. Due to significant ion pairing and a higher crystalline region (83% in Table 2) at 10 wt % loading, the ionic conductivity of the PAg composite reduces below the PCu system (Figure S5c). The PEO–metal NP composite films are excellent dielectrics. This is due to the complex relaxation capability with a variable frequency domain, as discussed in Section 3.4.3.

#### 3.4.3 Electrochemical Impedance and Dielectric Study of M^0^@PEO Composite Films

The Nyquist plot for the PEO–metal NP films is shown in Figure 15. Since the polymer composite relaxes through both ion hopping and segmental relaxation, the electrochemical reactions are impeded over a wide range of applied frequencies (1 Hz to 1 MHz). Irrespective of the type of composition, the bulk resistances (R1 and R2) along with a parallel fitted charge storage element (CPE) form a semicircle at higher frequency. This signifies the involvement of hopping charge carriers and macromolecular chain motion. The highest impedance is noted in the PEO matrix with Fe NPs (3.05–3.8 × 10^4^ Ω, Figure 16), as expected based on the ionic conduction trend. PAg_T_ and PAg_N_ are marked by the minimum nonlinear losses and are separately shown in a rugged plot in Figure 15b.^23^

**Figure 15:**
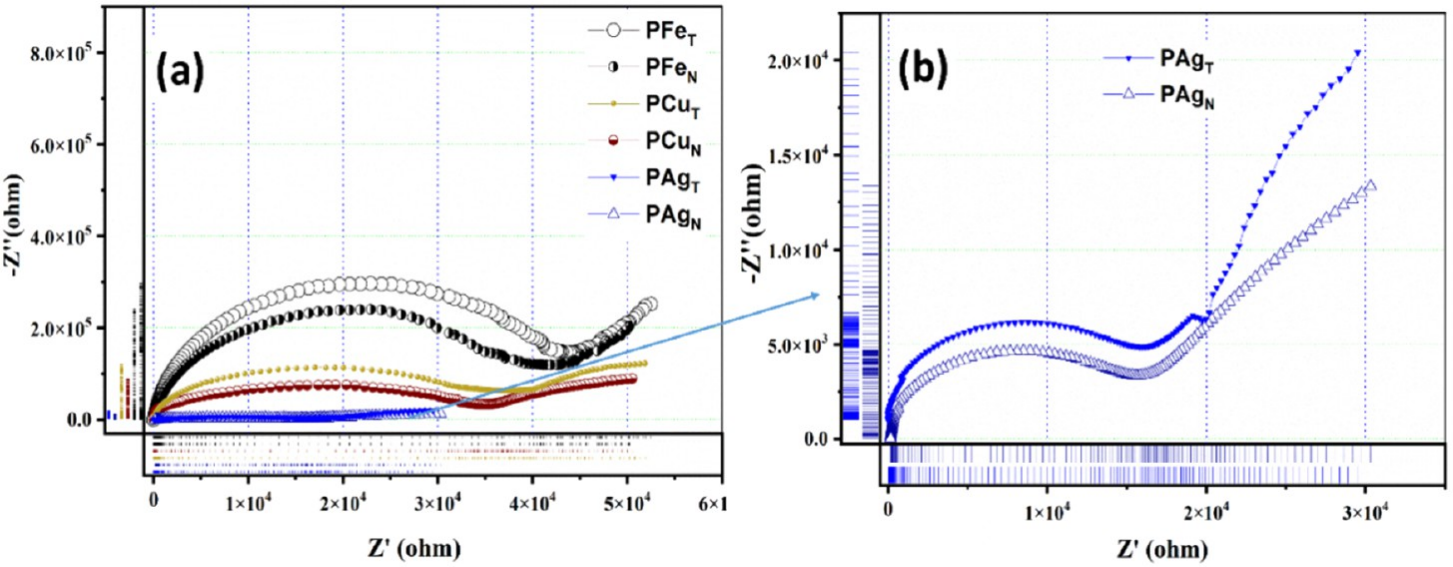
Nyquist plot obtained from EIS study for (a) PAg, PCu, and PFe series obtained from both neem and tea extract, and (b) zoomed plot for PAg_T_ and PAg_N_.

**Figure 16:**
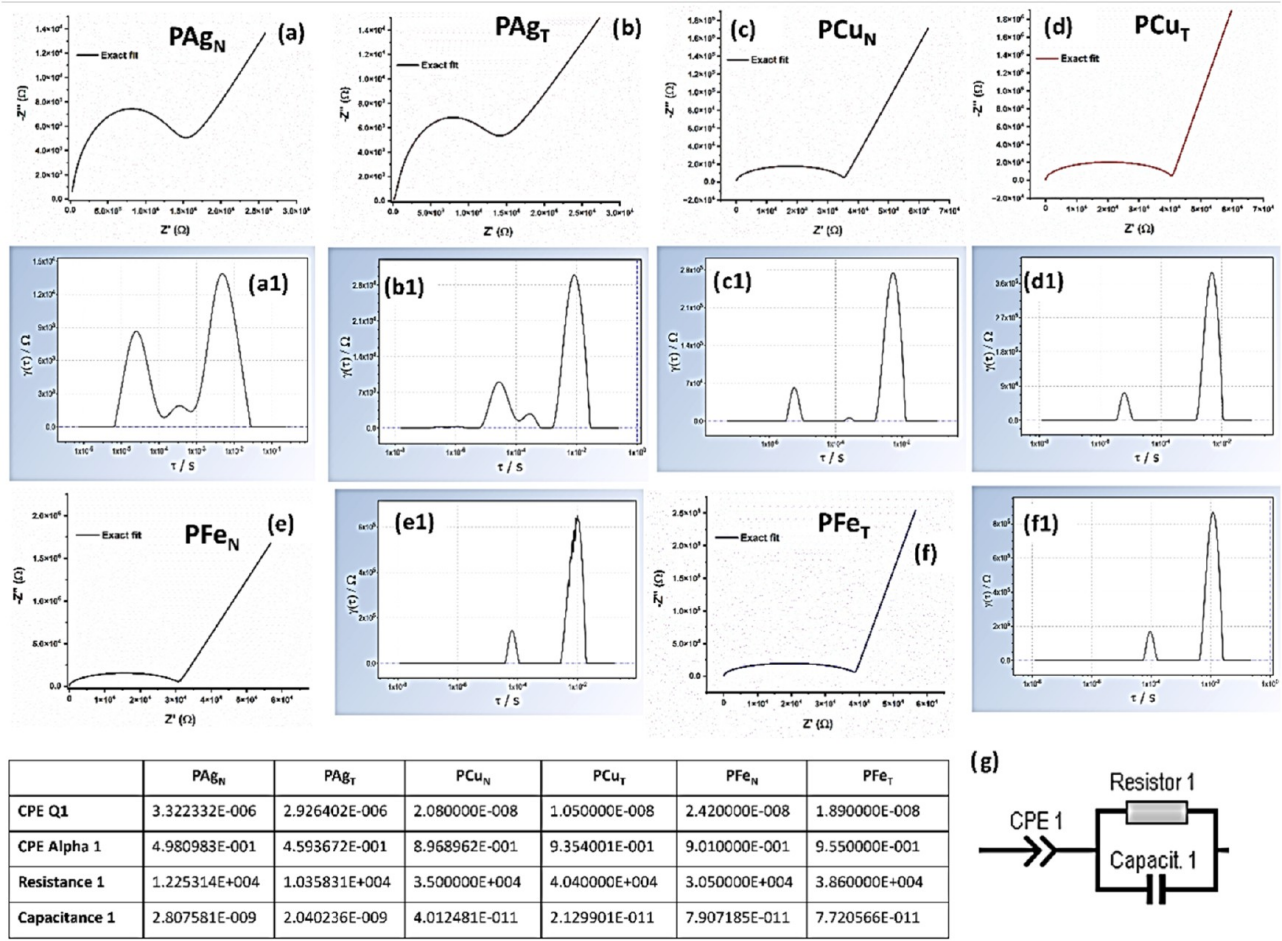
Representation of EIS fitting plots using an equivalent circuit (g) and distribution of relaxation time (DRT) for (a; a1) PAg_N_, (b, b1) PAg_T_, (c, c1) PCu_N_, (d, d1) PCu_T_, (e, e1) PFe_N_, and (f, f1) PFe_T_. The EIS fitting parameters for experimental films are mentioned in the table.

The formation of double-layer capacitance at the electrode–electrolyte interface signifies the dual phase of the PEO matrix^67,68^ and is further supported by the equivalent circuit used for EIS fitting (Figure 16g). The fitted EIS plots are shown in Figure 16a–f along with the distribution of relaxation time (DRT) plots (Figure 16a1–f1). The *x*-coordinate of DRT represents relaxation time, which signifies the polarization behavior with reference to physical processes. The low-frequency peaks (observed at higher relaxation times, τ) correspond to the characteristics of the parent macromolecular polymer. The influence of NPs could be resonated at the higher frequency domain, being responsible for ionic migration and dielectric responses. It is evident from DRT plots that the high-frequency process (at lower τ) is found to be spontaneous for PAg_N_ compared to PAg_T_. This agrees with the higher ionic conduction of PAg_N_ films (Figures 11 and 12) noted for Cu and Fe NPs. In contrast to PCu and PFe series, PAg films exhibit more than two relaxation processes shown in the DRT plot (∼10^–3^–10^–4^ s). The DRT plots signify the resonating frequency zones of relaxation as a function of film composition. The least resistive losses are observed for highly conductive PAg films (table in Figure 16). Details on EIS fitting are described in the Supporting Information. The low-frequency peak is observed to be the activation barrier irrespective of the type of embedded NPs (∼10^–2^s). Furthermore, the position of the low-frequency peak is found to be the representative signature of the host polymer, being responsive at a similar frequency domain irrespective of NPs. The concept of dielectrics signifies the stored electric potential within a fixed volume element under the influence of an electric field. Hence, it is dependent on the intrinsic polarizations (relaxation methods) of the system in response to variable frequencies. In PEO, the motion of the chain is responsive at lower frequencies (i.e., requires higher perturbation times, τ). In the present context, all of the compositions are observed to show a higher dielectric constant (ε′) at lower frequency (Figure 17a), followed by a frequency-independent zone. This may be attributed to the tendency of dipoles in polymeric samples to orient themselves in the direction of the applied field in the low-frequency range. With an increase in the applied frequency, the dipoles fail to reorient and remain unresponsive. PEO–metal NP films are unresponsive within 10^4^–10^6^ Hz, wherein the system fails to resonate with the alteration in field change.

**Figure 17:**
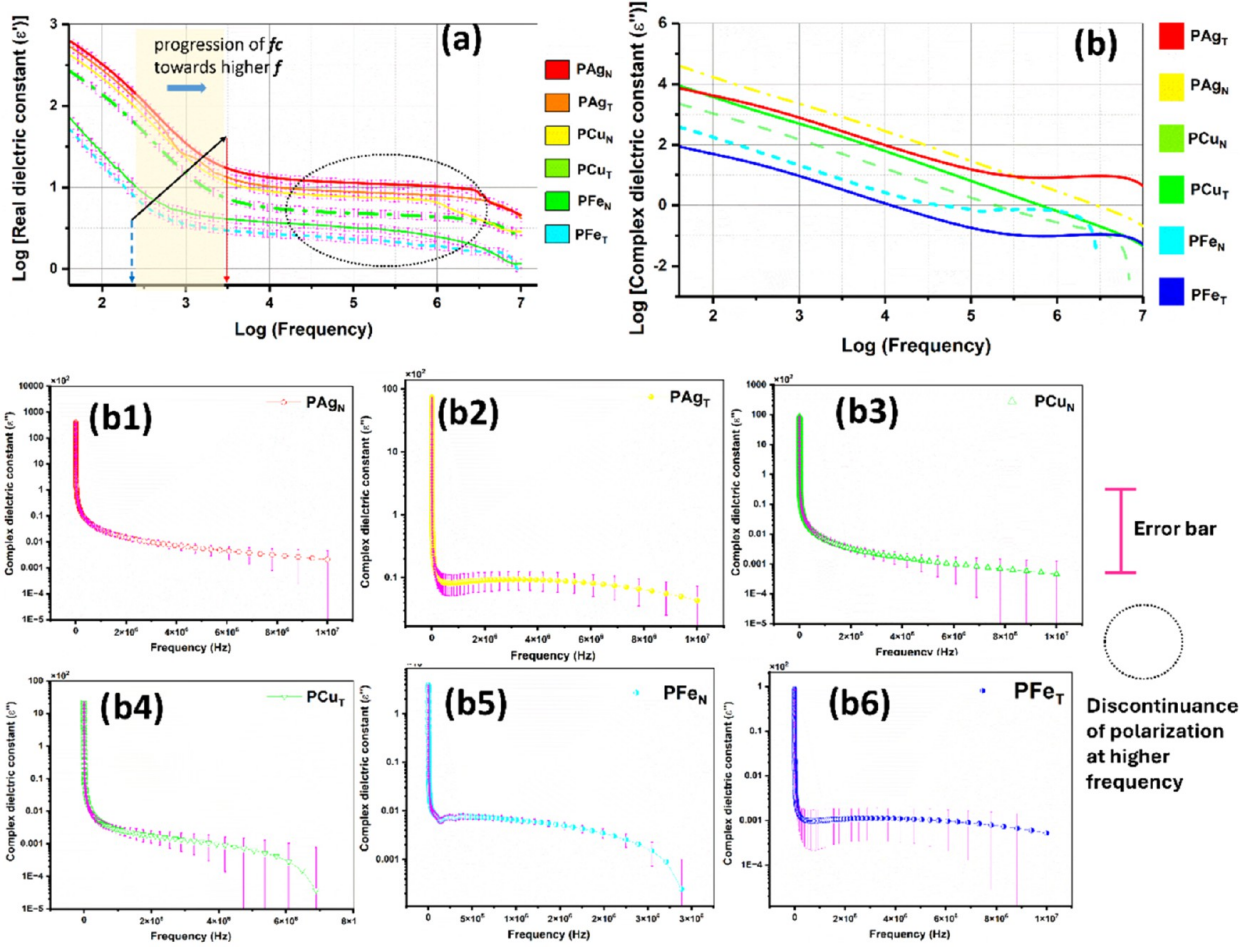
Variation of real (a) and complex (b1–b6) dielectric constants with error bars as a function of frequency for PAg, PCu, and PFe series. Influence of the phytoreductant on the dielectric loss (ε″) for (b1) PAg_N_, (b2) PAg_T_, (b3) PCu_N_, (b4) PCu_T_, (b5) PFe_N_, and (b6) PFe_T_.

This plateau zone signifies the relaxation of charge, which finally ceases at a higher frequency domain. The PAg system accounts for a higher dielectric constant owing to the generation of space charges at the electrode–electrolyte interfacial region. PAg_N_ contributes to the highest magnitude, and PFe_T_ contributes to the slowest counterpart of the dielectric constant. With progress in ionic conduction, the dielectric constant tends to increase with a shift in crossover frequency toward a higher dimension. This could be explained by the fact that the slower segmental motion of the host polymer composite makes it reorient at a much lower frequency. For Ag ions that are more labile and nanosized (evident from SEM), the extent of hopping is fast, and the associated secondary bonding with the PEO chain enables faster segmental motion. Consequently, the PAg matrix could be able to follow the higher frequency domain.

This could be correlated with the shift in crossover frequency (*f*_c_) toward higher magnitude with increased mobility of PEO chains (toward right, marked in Figure 17a). The crossover of the least conductive PFe_T_ having the lowest dielectric constant is observed to respond at a relatively lower applied frequency. The parameter of dielectric loss (ε″) has been reported to be more fundamental and is shown in Figure 17b. The nonlinear dependence of dielectric loss on the applied frequency (Figure 17b1–b6) shows an exponential reduction of ε″ with an increase in applied frequency for all PEO–metal NP compositions. The presence of mobile charges (ions) within the PEO matrix is a plausible factor for higher dielectric loss at a lower applied frequency. The net diffusion of ions is negligible with higher periodic reversal (at higher frequencies) in the applied direction of the electric field.^69,70^ It could be seen that the composite with higher ion conductivity (PAg_N_) also exhibited a higher dielectric loss, ∼10^6^ (Figure 17b1). Since the loss parameter signifies the transformation of electromagnetic energy into heat upon repeated dipolar orientation, a polymer matrix with higher charge transmittance responds to a higher complex dielectric parameter. PFe_T_ is observed to show a dielectric loss of ∼100 (Figure 17b6).

#### 3.4.4 Influence of Metal Nanoparticles on the Long-Term Cyclability of M^0^@PEO Composite Films

Loss tangent (tan δ) is a derived dimensionless parameter, which provides a measure of energy loss by a material when exposed to an electromagnetic field. For the macromolecules, tan δ can be computed using eq 7.

7 $$\tan\delta =\varepsilon^{\prime \prime }/\varepsilon^{\prime }$$

Figure 18a1–a3 shows the variation of tangent loss of all experimental PEO–metal NP films as a function of frequency. The peak in the tan δ plot signifies the relaxation of dipoles generated under the influence of reorganization of intrinsic polar species within the host polymer matrix, wherein the frequency is termed as relaxation frequency (*f*_relaxation_). The redistribution of charge is the intrinsic feature of the matrix, which tends to relax, subjected to secondary bond formation, charge transfer process, ion hopping, and ion pair generation.^71^

**Figure 18:**
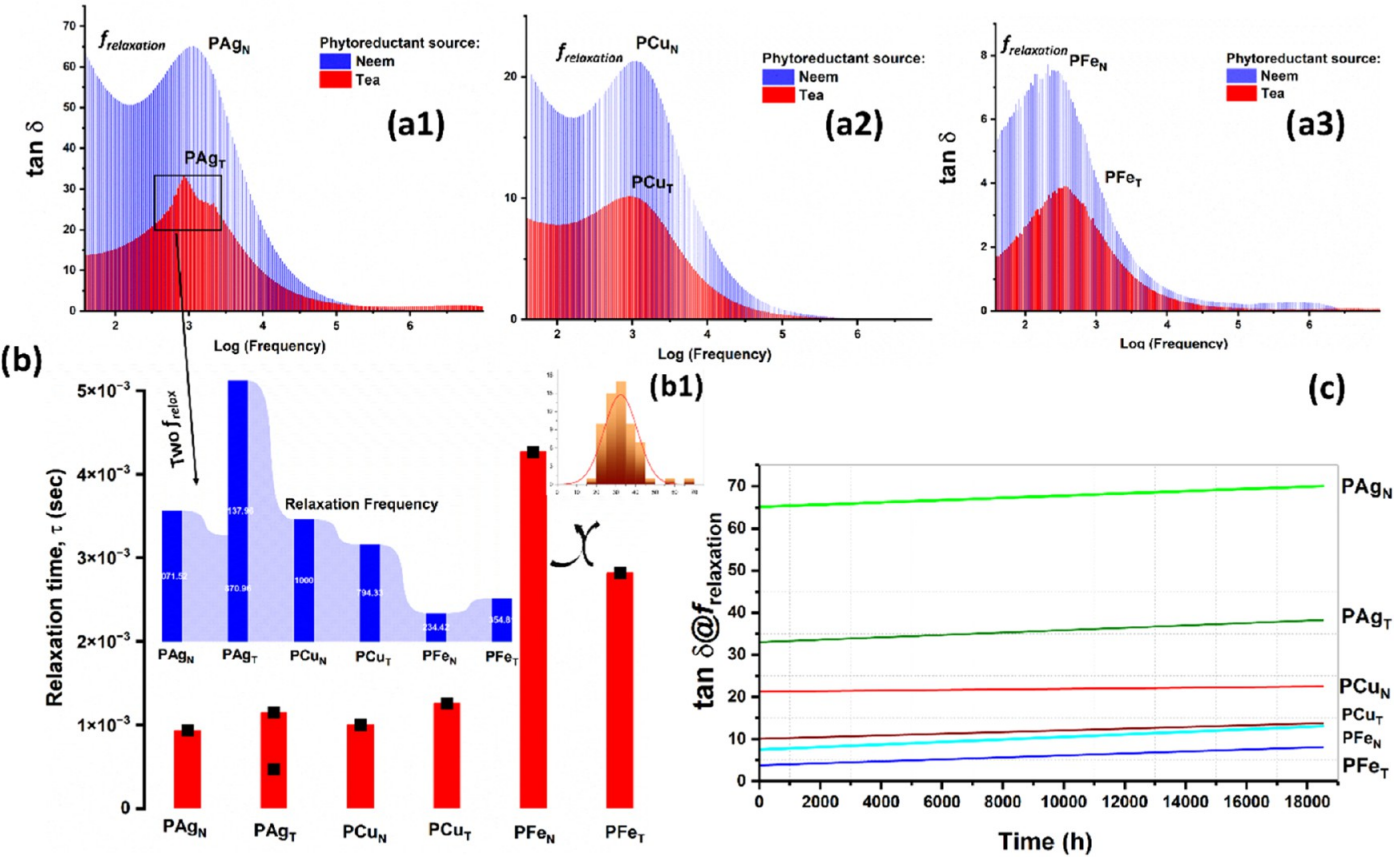
Variation of loss tangent (tan δ) as a function of field frequency for (a1) PAg, (a2) PCu, and (a3) PFe systems. (b) Correlation of morphology of metal NPs and PEO–metal NP composite with relaxation time required for matrix reorganization; (b1) inset particle size histogram of Fe NPs. (c) Endurance analyses of PAg, PCu, and PFe series films in terms of tan δ studied for 18,500 h.

The relaxation time (τ_relaxation_ = 1/*f*_relaxation_) is plotted as a function of polymer composition in Figure 18b. It could be noted that the compositions, PAg_N_, PCu_N_, and PFe_N_ synthesized using neem extract, tend to reorganize at higher relaxation frequencies. This could be linked with the higher ionic conduction of such matrices, thereby being capable of resonating with a higher applied field. PAg_T_ is observed to have two relaxation frequencies as marked in Figure 18b (in terms of τ_relax)_ and in the inset (in terms of *f*_relax_).

This could possibly reflect two relaxation processes to be operative in PAg_T_, which fail to convolute. PFe_N_ is observed to consume the maximum time for relaxation (>τ_relax_). This could be correlated with the highest degree of ripening of Fe NPs, resulting in a bigger particle size. The Fe NP upon dispersion within PEO tends to restrict the segmental motion of chains through secondary bonding. In addition, the hopping conduction within the PFe system is also lower compared to PAg and PCu systems (Figure 11a), which supports the formation of a bulky, slow-moving PEO composite system. In contrast, neem-derived PAg_N_ and PCu_N_ show a fast response to the applied frequency alteration and thereby perturb at comparatively higher frequencies (Figure 18a1). The relaxation behavior of the polymer composite with variation in NP doping is shown in Figures S6–S9. The trend observed for 1–3 wt % NP doping is found to be as expected, since higher loading of NPs tends to make the system less mobile, which requires a higher time to relax and respond at a lower perturbation frequency. However, Figures S8 and S9 signify the contribution of morphology, as discussed above. Higher doping of metal NPs enhances the crystalline zone and favors intraparticle ripening. In addition, ion pairing is significant, especially in the PAg matrix, which is observed to be bulky and requires the maximum time to relax at 10 wt % doping.

The long-term endurance of the synthesized PEO–metal NP composite films was studied for 18,500 h and was represented in terms of tangent loss. At a particular time interval, tan δ is selected at the relaxation frequency (*f*_relaxation_), so that maximum reorganization at an applied frequency is achieved. In addition, the magnitude of relaxation frequency is an intrinsic property of the polymer matrix, which ensures the contribution of the tailored morphology of the PEO–metal NP. It could be noted that with time, the magnitude of tan δ@f_relax_ increases, and the increment is unique with respect to the parent matrix. Among all of the compositions, PCu_N_ is observed to exhibit negligible deterioration, which corroborates the finding in Figure 11c. The labile redox chemistry of the Cu^2+^/Cu couple ensures optimum distribution of particle size on NPs, which suffers the least ripening compared to Ag and Fe, respectively. However, the PAg system exhibits the highest ion conduction; however, the associated performance deterioration is maximum (Figure 18c). The present study reports two aspects of application for PEO–metal NP films. Based on the optimization of NP size and morphology, the ion conduction of the PEO matrix is observed to be maximum for Ag-based nanocomposite films, which show excellent dielectric storage as well. However, for long-term cyclability, the PAg system demands a compromise in terms of performance deterioration. In contrast, the Cu nanoparticles undergo ripening (since Cu^2+^/Cu kinetics is labile) and are less conductive at the operating temperature. The PCu system, however, does not show any performance degradation. Hence, green synthesis offers an effective, simple technique for the fabrication of PEO-based films that are excellent ion conductors with significant dielectric storage capability.

The reported PEO composite films find potential application as polymer electrolytes in solid-state devices.^9–11^ The lightweight feature with excellent endurability is an advantage of these systems for potential application within devices. In addition, owing to significant dielectric properties and ion migration capabilities, these could find usage in the biomedical field. However, the biocompatibility needs to be assessed as per the set protocols.

## 4 Conclusions

In a nutshell, the present study reports excellent endurability (18,500 h) of poly­(ethylene oxide) (PEO)-based films that are functionalized by metal nanoparticles (NPs) such as Fe^0^, Cu^0^, and Ag^0^. Metal NPs are produced using green synthesis using phytochemicals from herbs such as *Camellia sinensis* (Tea) and *Azadirachta indica* (Neem). The novelty of the present study lies in the prediction of a plausible mechanism for the green synthesis of metal nanoparticles (Ag^0^, Cu^0^, and Fe^0^) using density functional theory (DFT). Furthermore, along with enhanced endurance, the ion conduction (0.1 S cm^–1^ for PEO–Ag^0^ films) and dielectric storage could be tailored as a function of metal NPs using a specific selection of phytoreductants and capping agents. It has been studied that formation of metal NPs involves an initial activation, wherein selected phytochemicals act as reducing agents. This is followed by nucleation of NPs and termination through capping (complementary phytochemicals), which restricts Ostwald ripening to form Ag^0^ (*d*_p_ ∼ 6 nm), Cu^0^ (*d*_p_ ∼ 25 nm), and Fe^0^ (*d*_p_ ∼ 35 nm). The reducing and/or capping agents functionalize through −OH and involve the tautomeric mode of stabilization of the metal NP–bioreagent complex. Density functional theory (DFT) was engaged in this regard to study the energetics of all known phytochemicals of tea and neem. The energy change (band gap, Δ*E*_|LUMO–HOMO|_) is found to be minimum for Epicatechin gallate (tea) and Sitosterol (neem), which could act as potent phytoreductants for initiating a redox reaction, followed by subsequent capping through secondary bond formation. Formation of metal NPs was ascertained by UV–vis spectroscopy (in the solution phase) and X-ray diffraction (powder phase). The % crystallinity of the PEO–metal NP films is evaluated using XRD as well as differential scanning calorimetry (DSC). Among possible loadings, 7 wt % of metal NP loading was optimized to maximize the ion conduction of PAg_N_ (P: polymer, N: neem) to 0.1 S cm^–1^@room temperature. A further increase in metal NP loading to 10 wt % was studied to enhance the % crystallinity to 83% (PAg), 81% (PCu), and 80% (PFe). As is evident, the ionic conduction and charge storage capacity of phase-pure PEO–metal NP are reduced at 10 wt % loading. This is also corroborated by the scanning electron morphology. Among all of the compositions, PCu_N_ is observed to exhibit negligible deterioration in ion conductivity per 1000 h and performance (tangent δ for 18,500 h), which corroborates with the intrinsic least tendency of ion pairing. The PAg system(s) are excellent ion conductors with significant dielectric storage but suffer from ion pairing and particle ripening. This is influenced by a comparatively higher deterioration rate of tangent loss (tan δ) over 18,500 h. The authors studied the complex dielectric relaxation of all compositions as a function of metal NP doping. As expected, the higher ripening tendency of Fe NPs (studied using SEM and PSD) results in a much higher relaxation time (τ). The relaxation process is studied in detail using the distribution of relaxation time (DRT), which shows signatures of a macromolecular polymer chain at lower perturbation frequencies (∼10^–2^ s). The high-frequency DRT peaks are, however, influenced by the metal NPs and could be tailored to control the ion migration and dielectric responses. However, for Ag/Cu NPs doped for 1–7 wt %, the ions are mobile and could cause significant segmental motion of the polymer chain. Therefore, compared to using inorganic salts in prior studies, metal NPs synthesized using a green synthesis route act as potent charge carriers within the polymer host and increase the shelf life of the electrolyte. Hence, phytoreduction of M^*n*+^ could be effectively controlled through green synthesis for functionalization of the host polymer matrix.

## Supplementary Material



## Notes

1) Novelty of the present research with comparative prior studies; 2) Details of selected bioactive agents in *Camellia sinensis* and *Azadirachta indica* along with their standard reduction potentials; 3) Theoretical study on the bioactive agents of *Camellia sinensis* and *Azadirachta indica*; 4) AC ion conduction in PEO nanocomposite films using EIS for 1, 3, and 10 wt % NP loading; complex relaxation of PEO–metal NP films; 5) Dependence of relaxation time for PEO–metal NP films as a function of metal NP loading; and 6) Electrochemical impedance spectra—equivalent circuit fitting (PDF)
