# Supplementary material for: Green Chemistry-Assisted Synthesis of Metal Nanoparticles and Fabrication of Microstructurally Engineered Conductive and Endurable M0@PEO Functional Films
Source: ACS Omega. 2025 Aug 19;10(34):38609–28. doi: 10.1021/acsomega.5c03323 (PMC12409529; doi:10.1021/acsomega.5c03323)
Supplement: Supplementary file 1 [file ao5c03323_si_001.pdf]

Green Chemistry-assisted Synthesis of Metal Nanoparticle and Fabrication of Microstructurally Engineered Conductive and Endurable M^{o}@PEO Functional Films

Anamika Das,[a,b] *Raktima Chatterjee,*[c,d#] *Shinjini Sarkar,*[e#] *Grishma Ninave,*[e] *Debosreeta Bose,*[e] *Amit Kumar Dutta,*[f] *Satarupa Biswas,*[b] *Moumita Mukherjee,*[b] *Ragavendran Venkatesan,*[g] *Rahul Majee,*[h] *Saumya Dasgupta,** *Jayanta Mukhopadhyay**[, i, d] *and Madhumita Mukhopadhyay**[,b]

[#]Contributed equally

[a]Department of Physics, Acharya Prafulla Chandra College, New Barrackpore, Kolkata, West Bengal 700131, India

[b]Department of Physics, Adamas University, Kolkata - Barrackpore-Barasat Road, Kolkata, West Bengal 700126, India

[c]Specialty Glass Division, CSIR-Central Glass & Ceramic Research Institute, Jadavpur, Kolkata – 700032, West Bengal, India

[d]Academy of Scientific and Innovative Research (AcSIR), Ghaziabad, Uttar Pradesh, India

[e]Department of Chemistry, Amity Institute of Applied Sciences (AIAS), Amity University, Kolkata-700156, West Bengal, India

[f]Department of Chemistry, Bangabasi Morning College, 19 Raj Kumar Chakraborty Sarani, Kolkata – 700009, West Bengal, India

[g]Department of Physics, Rajalakshmi Engineering College, Thandalam, Chennai-602105, Tamil Nadu, India

[h]School of Chemistry, University of St Andrews, St Andrews, Fife, KY16 9ST, United Kingdom

[i]Energy Materials & Devices Division, CSIR-Central Glass and Ceramic Research Institute, Kolkata, West Bengal, India

*Corresponding Author (s)

• madhubanerji@gmail.com & mm613@st-andrews.ac.uk (MM) • jayanta_mu@cgcri.res.in & jmukhopadhyay75@gmail.com(JM); • sdasgupta@kol.amity.edu (SDG)

Supporting Information

1. Novelty of present research with comparative prior art (s)

Table S1. Comparative study on the novelty of present research with respect to prior art literature. [S1-S6]

Sl No	Polymer composite films reported in literature	Novelty of the research
1.	Present research:<br>Ag°@PEO, Cu°@PEO, Fe°@PEO	• Green synthesis route [extracts of *Camellia sinensis* (Tea) *and Azadirachta indica* (Neem)] utilized for synthesis of M°<br>• Ionic conductivity: Ag°@PEO (0.1 $S.cm^{-1}$), Cu°@PEO (0.045 $S.cm^{-1}$), Fe°@PEO (0.018 $S.cm^{-1}$)<br>• DFT utilized to establish Epicatechin gallate (tea) and Sitosterol (neem) as the potential phyto-reductant for M°<br>• Dielectric constant of 1000 for M°@PEO which signifies good electrical energy storage capacity<br>• 18500 h endurance study with negligible tangent loss
2.	PEO/ AgNPs-PVP films<br>Ref. S1: Journal of Inorganic and Organometallic Polymers and Materials 2024, 35 (2), 1281–1291	• Ag NPs are synthesized by chemical means<br>• Polyethylene oxide (PEO) and various concentrations of Ag NPs coated with polyvinylpyrrolidone (PVP) are reported<br>• XRD, DSC and UV are reported to characterize the film
		• AC conductivity of PEO-CMC with Cu NPs (1.6 wt. %) is 3.71×10^{-6} $S.cm^{-1}$

3	PEO/CMC-Cu nanocomposites<br><br>Ref. S2: Optical Materials **2022**, 134, 113092.	• Dielectric constant value for nanocomposite with highest concentration is 6.86 × 10^4 at 0.1 Hz which is near the minimum required to qualify as Super Dielectric Material
4	PEO-Zein NPs<br><br>Ref. S3: Colloids and Surfaces A: Physicochemical and Engineering Aspects 2020, 586, 124268	• Zein NPs- Chemical synthesis<br>• Film was characterized for Antioxidant activity
5	Ultrathin PEO based electrolyte for Li metal batteries<br><br>Ref. S4: Journal of Energy Storage 2023, 68, 107640–107640	• Excellent ionic conductivity of 0.58 mS cm^{-1} at 30 ◦C<br>• High Li^+ transference number of 0.79 and improved electrochemical window of 5.2 V<br>• Li|DSPE|Li battery can be cycled stably for 900 h at 0.1 mA cm^{-2}
6	ZnO NP-PEO based Solid-State Lithium−Polymer Batteries<br><br>Ref. S5: The Journal of Physical Chemistry C **2020**, 124 (51), 27907–27915	• ZnO NPs – chemical synthesis route<br>• 4.2 × 10^{-4} S cm^{-1} at room temperature, which increases to7.1 × 10^{-3} S cm−1 at 80 °C (with 7 wt. % ZnO)
7	PEO/TCPP/$LiClO_4$ SPE<br><br>Ref. S6: Journal of Physical Chemistry C 2021, 125 (42), 22960–22969	• First time report to insert TCPP – COF as filler within energy storage system<br>• Green solution cast technique is employed

2. Details of selected bioactive agents in *Camellia sinensis* and *Azadirachta indica* along with their Standard reduction potentials

Table S2: Salient Bioactive agent in *Camellia sinensis* and *Azadirachta indica* along with their Standard reduction potentials (vs. SHE at 37 ± 0.1 ˚C)

**More than one inflection points on the titration curve are marked as 1st infl. and 2nd inf*

Herb family	Bioactive agent	Structure	DFT optimized structure	Standard reduction potential, E^o (V) /Cyclic Voltammogram
	Catechin	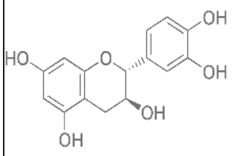	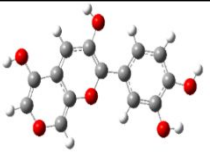	0.281 ± 0.008 [30] ;
	Catechin gallate	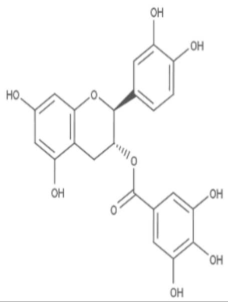	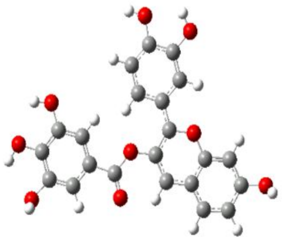	Alike Epicatechin gallate [30]
Camellia sinensis	Epicatechin	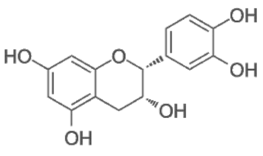	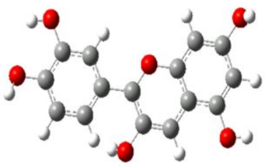	0.277 ± 0.005 [30]

(Tea)	Epicatechin gallate	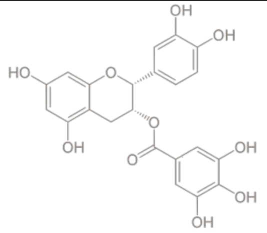	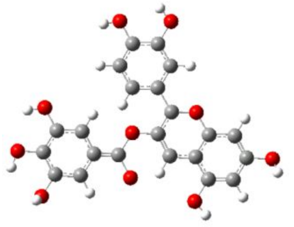	• 0.098 ± 0.002 (1st infl.)<br>• 0.146 ± 0.003(2nd infl.) [30]
	Epigallocatechin	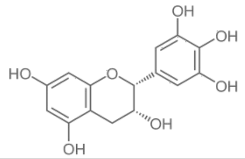	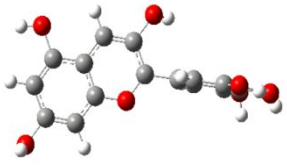	0.287 ± 0.003 [30]
	Epigallocatechin gallate	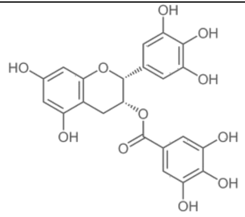	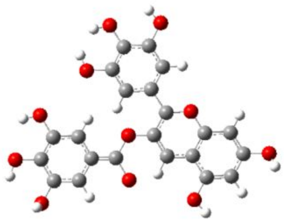	• 0.104 ± 0.002 (1st infl.)<br>• 0.153 ± 0.001 (2nd infl.) [30]
	Theaflavin	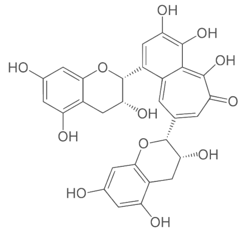	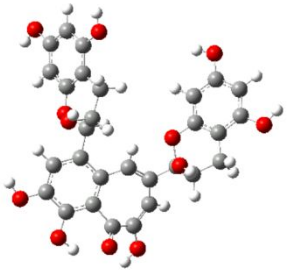	0.48- 0.54 [31]
	Theaflavin-3-gallate	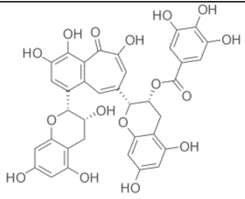	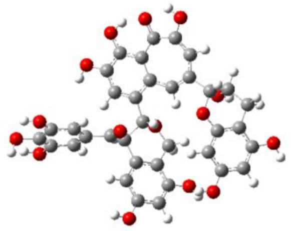	0.54 [31]
	Azadirachtin	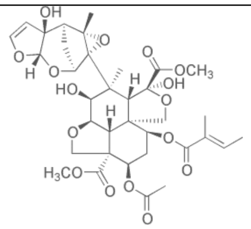	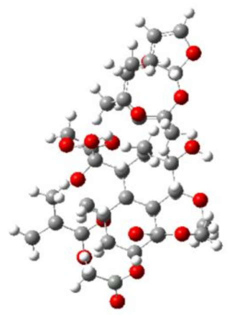	1.48 [32]
Azadirachta indica (Neem)	Nimbin	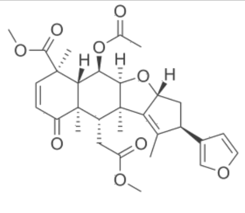	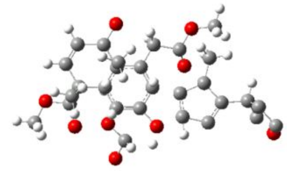	Multiple oxidation process to produce nimbinolide and isonimbinolide involving oxygen containing species. [33]

	Nimbolin A	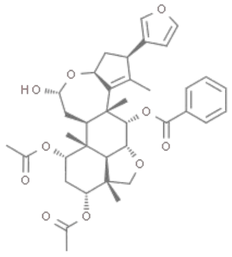	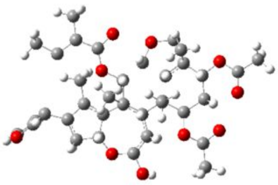	The oxidation involves conversion of its secondary alcohol group into a ketone [33]
	Quercetin	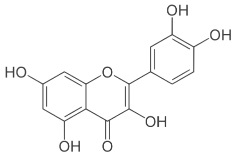	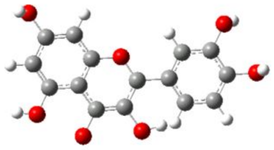	Multistep Oxidation products involving potentials (determined from CV) Ouinone intermediate (+0.35 V), cleavage products, simple phenolics, Depsides, Open structures, Benzofuranoes (+0.8 V), Oligomers and polymers. [34]
	Sitosterol	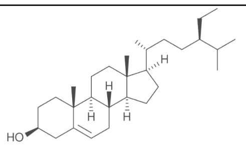	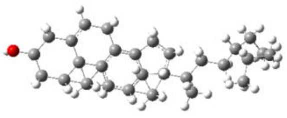	The oxidation current appeared at >+ 1.8 V vs. Ag/AgCl and the oxidation peak was around 2.9 V vs. Ag/AgCl. [35]

3. Theoretical study on bioactive agents of *Camellia sinensis* and *Azadirachta indica*

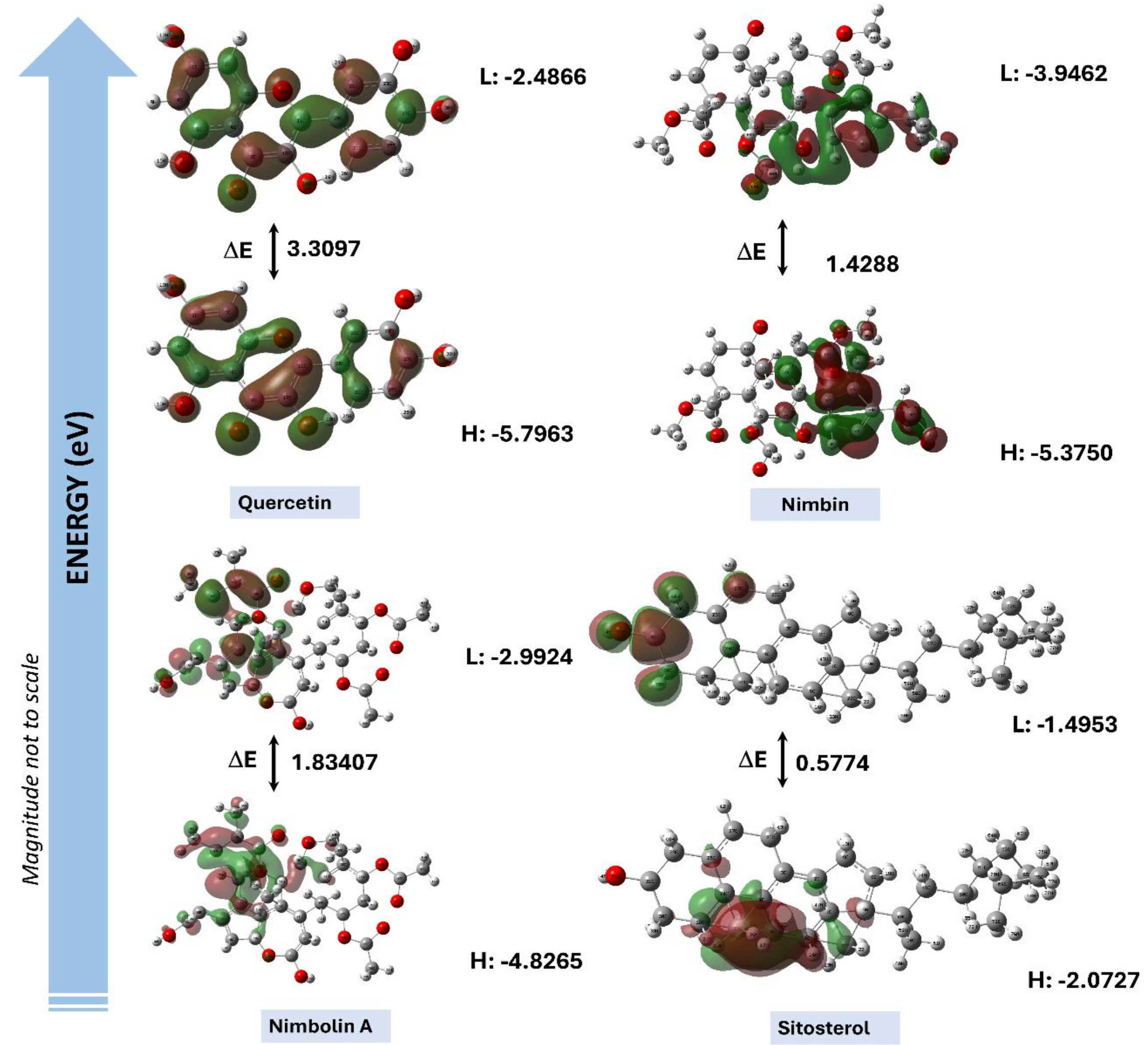

Figure S1. Molecular orbital (using DFT) representation for Phyto reductants obtained from *Azadirachta indica* [H: HOMO & L: LUMO; Energy is in eV]

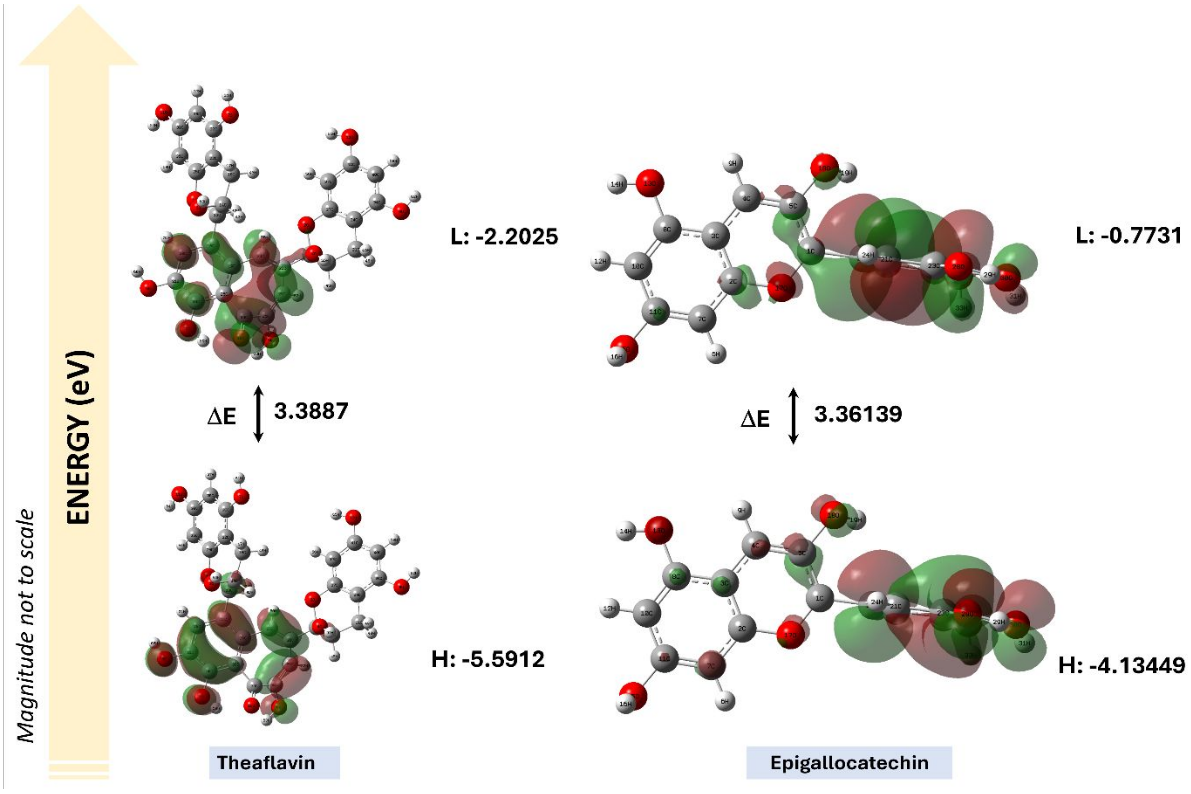

Figure S2. Molecular orbital (using DFT) representation for Phyto reductants (Theaflavin and Epigallocatechin) obtained from *Camellia sinensis* [H: HOMO & L: LUMO, Energy is in eV]

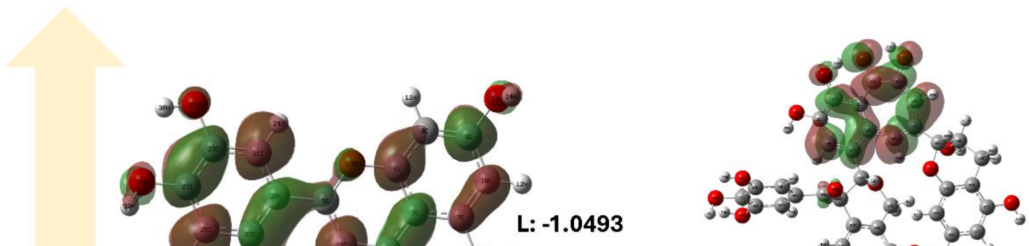

Figure S3. Molecular orbital (using DFT) representation for Phyto reductants (Epicatechin and Theaflavin-3-Gallate) obtained from *Camellia sinensis* [H: HOMO & L: LUMO, Energy is in eV]

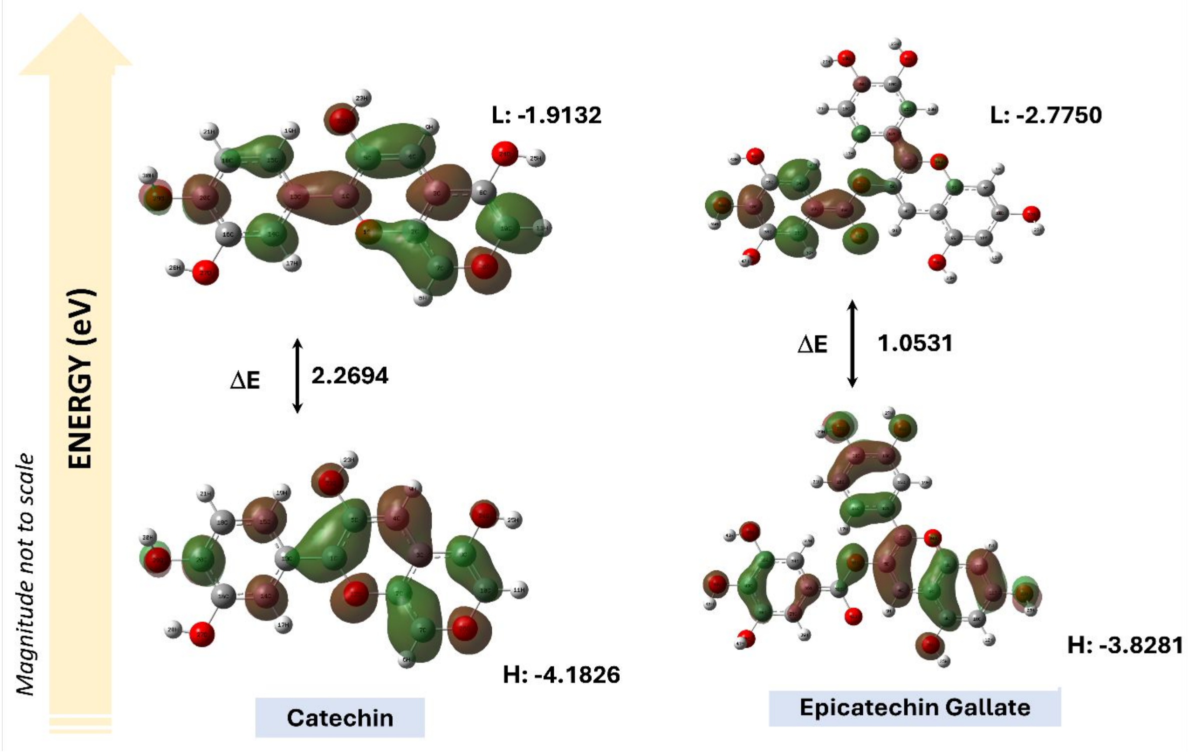

Figure S4. Molecular orbital (using DFT) representation for Phyto reductants (Catechin and Epicatechin Gallate) obtained from *Camellia sinensis* [H: HOMO & L: LUMO, Energy is in eV]

4. AC ion conduction in PEO-nanocomposite films using EIS for 1, 3 and 10 wt. % NP loading

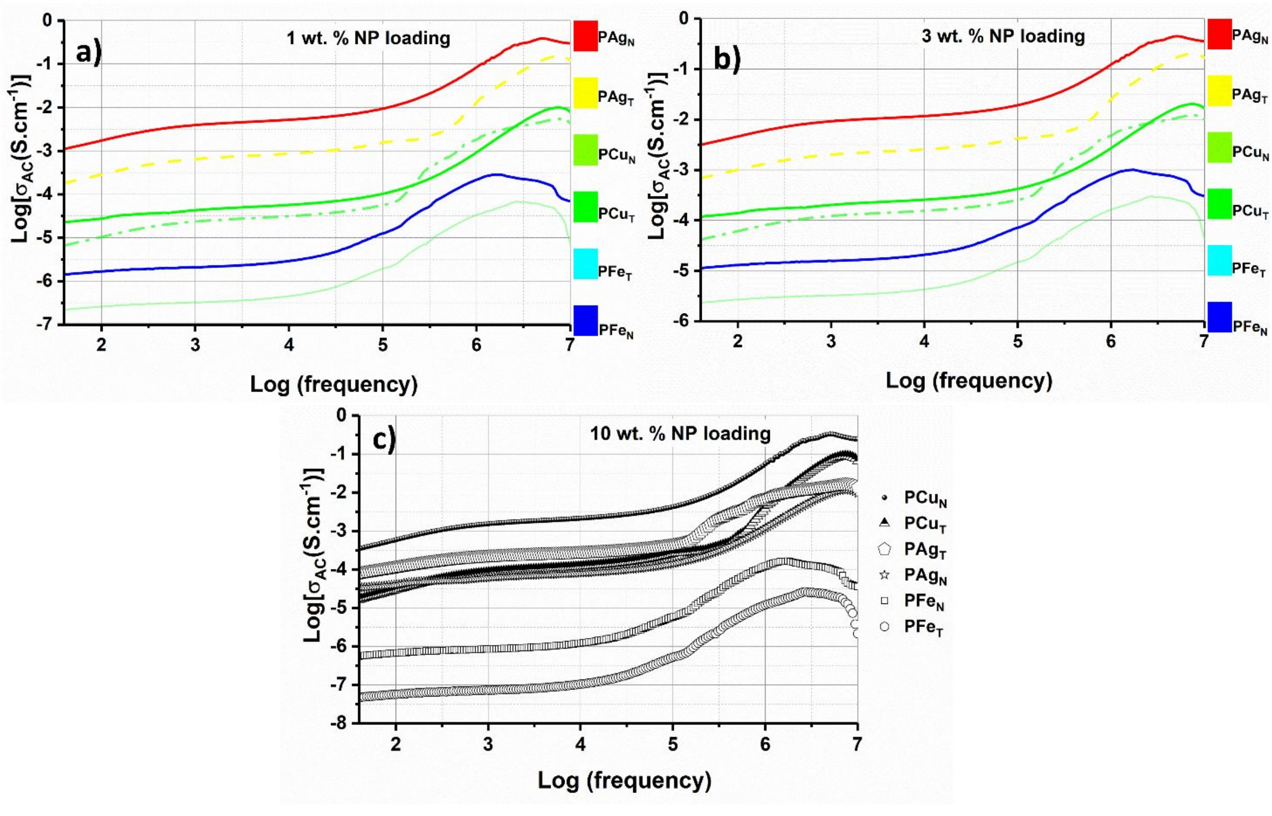

Figure S5. Frequency dependent AC conductivity as a function of PEO-Metal NPs composite films with respect to NP doping of: a) 1 wt. %, b) 3 wt. % and c) 10 wt. %,

5. Complex relaxation of PEO-metal NP films

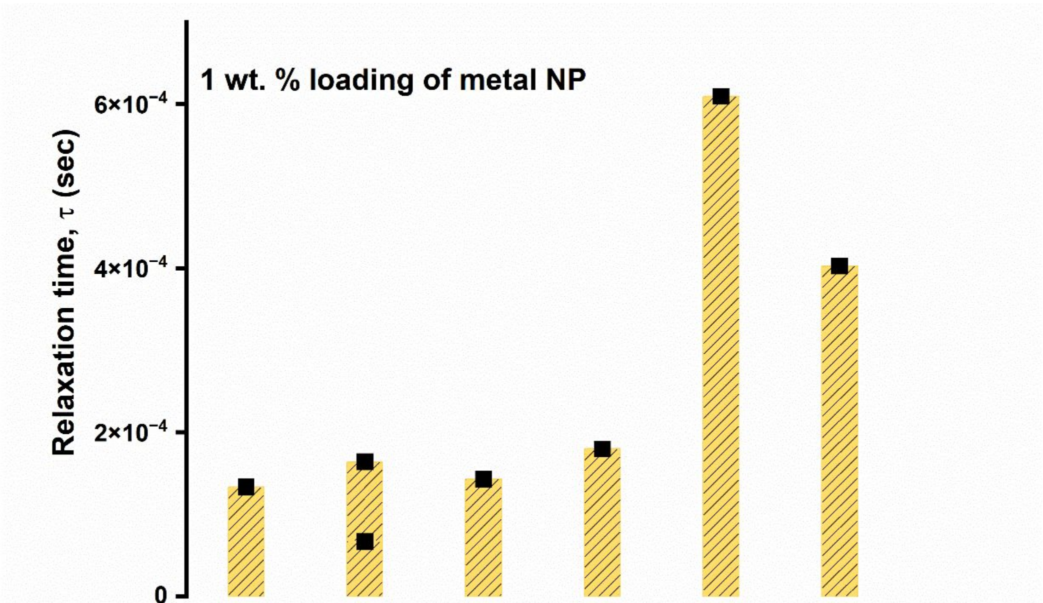

Figure S6. Trend of relaxation time for PEO-metal NP films for 1 wt. % doping of NP

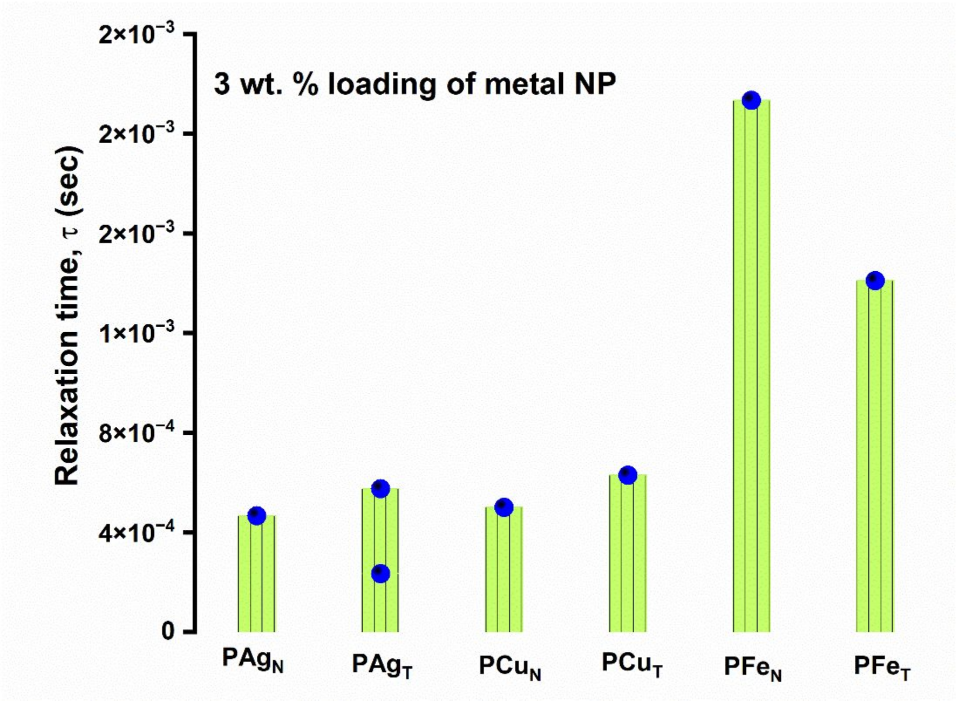

Figure S7. Trend of relaxation time for PEO-metal NP films for 3 wt. % doping

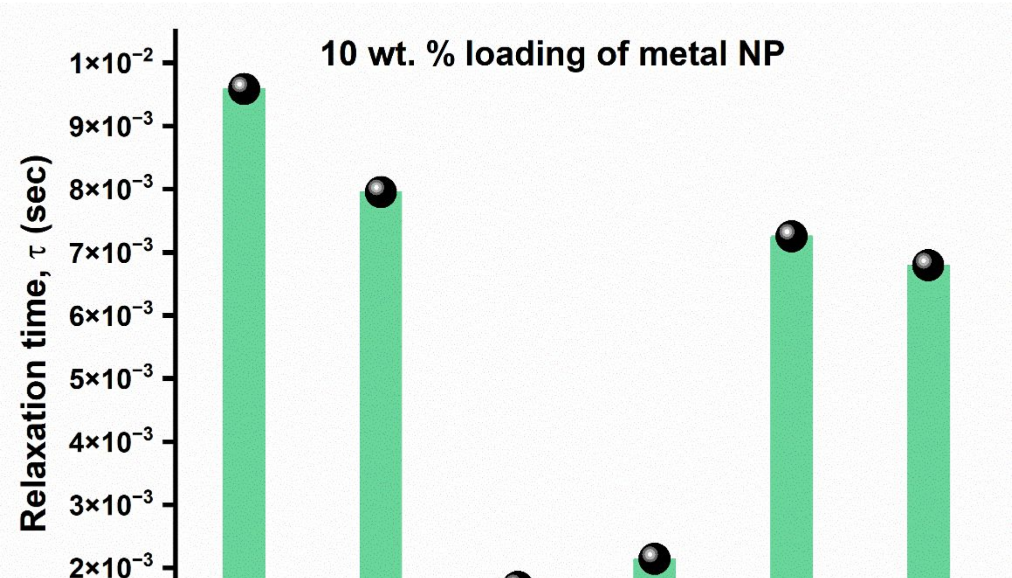

Figure S8. Trend of relaxation time for PEO-metal NP films for 10 wt. % doping of NP

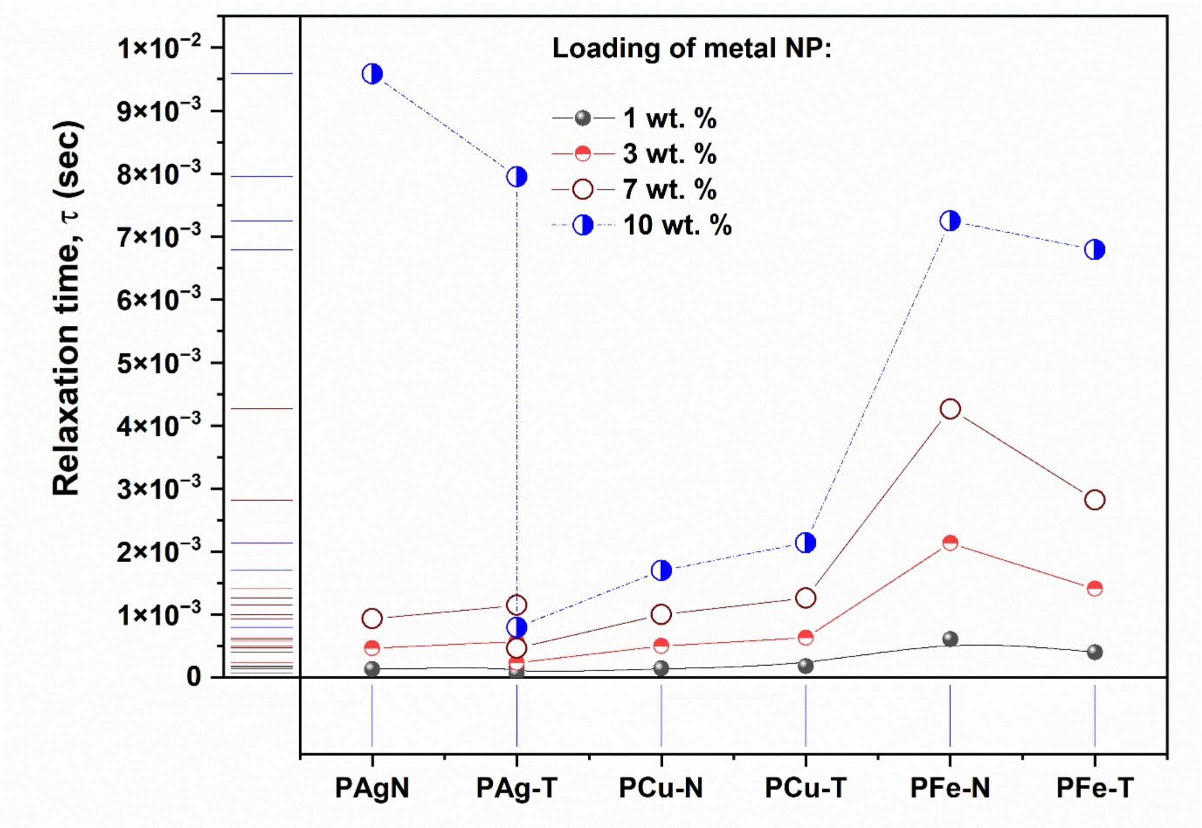

Figure S9. Dependence of relaxation time for PEO-metal NP films as a function of metal NP loading

6. Electrochemical Impedance spectra- Equivalent circuit fitting

Fit Details

A. <u>Datasource: PAgN.txt_1</u>

Model: P-(R)(C)

n Datapoints: 132 (264 total dependant values)

Free parameters: 4

DOF: 260

Last transfer function: Admittance

Last weight mode: Proportional Weighting

Covariance matrix:

Correlation matrix:

Fitparameters (from last fit)

CPE Q 1 : 3.322332E-006 (+- 6.190704E-007)

CPE Alpha 1 : 4.980983E-001 (+- 2.820808E-002)

Resistance 1 : 1.225314E+004 (+- 6.278836E+002)

Capacitance 1: 2.807581E-009 (+- 1.822088E-010)

Statistics for: Impedance

SSR: 679108854.699455 (Weighted: 71.891041753171)

Chi²: 2611957.13345944 (Weighted: 0.276504006742965)

SEOE: 1603.86471542232

AIC (prop.): 5109.83471986725

R²: Nonlinear: 0.979350027909506, Linear: 0.94336232436255

Adj. R²: Nonlinear: 0.979032336031191, Linear: 0.942708812720579

Statistics for: Admittance

SSR: 0.000104697106335808 (Weighted: 11.7440111914962)

Chi²: 4.02681178214645E-07 (Weighted: 0.0451692738134468)

SEOE: 0.000629745939440419

AIC (prop.): -4756.57405186081

R^2: Nonlinear: 0.435320589117623, Linear: 0.335099402962012

Adj. R^2: Nonlinear: 0.426633213565587, Linear: 0.327427472996189

Statistics for: Complex Capacitance

SSR: 3.01507502041776E-15 (Weighted: 11.7440111914962)

Chi²: 1.15964423862222E-17 (Weighted: 0.0451692738134469)

SEOE: 3.37945834620042E-09

AIC (prop.): -9823.96150984296

R^2: Nonlinear: 0.982603013545523, Linear: 0.973739880014899

Adj. R^2: Nonlinear: 0.982335367600069, Linear: 0.973436878630456

Statistics for: Elastance

SSR: 2.64519693007387E+18 (Weighted: 71.891041753171)

Chi²: 1.0173834346438E+16 (Weighted: 0.276504006742965)

SEOE: 100098378.314481

AIC (prop.): 10177.2221778494

R^2: Nonlinear: 0.629655992603983, Linear: 0.18447590826435

Adj. R^2: Nonlinear: 0.623958392490198, Linear: 0.17506601489817

B. Data source: PAgT.txt_1

Model: P-(R)(C)

n Datapoints: 194 (388 total dependant values)

Free parameters: 4

DOF: 384

Last transfer function: Impedance

Last weight mode: Proportional Weighting

Fitparameters (from last fit)

CPE Q 1 : 2.926402E-006 (+- 4.834929E-007)

CPE Alpha 1 : 4.593672E-001 (+- 1.650157E-002)

Resistance 1 : 1.035831E+004 (+- 7.471349E+002)

Capacitance 1: 2.040236E-009 (+- 1.564767E-010)

Statistics for: Impedance

SSR: 264410697.291363 (Weighted: 40.3220349984983)

Chi²: 688569.524196259 (Weighted: 0.105005299475256)

SEOE: 825.512488886532

AIC (prop.): 6815.10318308653

R²: Nonlinear: 0.99260876811933, Linear: 0.986492075220218

Adj. R²: Nonlinear: 0.992531776120573, Linear: 0.986386544557875

Statistics for: Admittance

SSR: 0.000847850960430817 (Weighted: 889.957858712078)

Chi²: 2.20794520945525E-06 (Weighted: 2.31759859039604)

SEOE: 0.00147823641130514

AIC (prop.): -5149.27870735652

R^2: Nonlinear: -13.1620896439833, Linear: -26.817613067561

Adj. R^2: Nonlinear: -13.3096114111081, Linear: -27.0349381696513

Statistics for: Complex Capacitance

SSR: 7.62664400247227E-16 (Weighted: 889.957858712079)

Chi²: 1.98610520897715E-18 (Weighted: 2.31759859039604)

SEOE: 1.40200923589839E-09

AIC (prop.): -14091.7888700471

R^2: Nonlinear: 0.995090618756403, Linear: 0.993678249505232

Adj. R^2: Nonlinear: 0.995039479368449, Linear: 0.993628860829492

Statistics for: Elastance

SSR: 1.16247967859845E+22 (Weighted: 40.3220349984983)

Chi²: 3.02729082968348E+19 (Weighted: 0.105005299475256)

SEOE: 5473647454.73465

AIC (prop.): 15757.6133457771

R^2: Nonlinear: 0.203916659640913, Linear: 0.0271281254956299

Adj. R^2: Nonlinear: 0.195624124845506, Linear: 0.0195275639760646

C. Datasource: PCuN.txt_1

Model: P-(R)(C)

n Datapoints: 163 (326 total dependant values)

Free parameters: 2

DOF: 324

Last transfer function: Impedance

Last weight mode: Proportional Weighting

Fitparameters (from last fit)

CPE Q 1 : 2.080000E-008 (+- 0.000000E+000)

CPE Alpha 1 : 8.968962E-001 (+- 9.076598E-003)

Resistance 1 : 3.500000E+004 (+- 0.000000E+000)

Capacitance 1: 4.012481E-011 (+- 3.389805E-012)

Statistics for: Impedance

SSR: 183890696473.064 (Weighted: 48.8227043421494)

Chi²: 567563878.003285 (Weighted: 0.150687359080708)

SEOE: 23750.4082285267

AIC (prop.): 7199.39263496505

R²: Nonlinear: 0.658460112053817, Linear: -0.306544872778795

Adj. R^2: Nonlinear: 0.656351841140569, Linear: -0.310577418682433

Statistics for: Admittance

SSR: 2.12364981494057E-07 (Weighted: 146.929305089447)

Chi²: 6.5544747374709E-10 (Weighted: 0.453485509535332)

SEOE: 2.55230547466678E-05

AIC (prop.): -6215.87795475822

R^2: Nonlinear: 0.81909485765297, Linear: 0.748181553776915

Adj. R^2: Nonlinear: 0.81797815924342, Linear: 0.747404336350301

Statistics for: Complex Capacitance

SSR: 3.8702299306283E-16 (Weighted: 146.929305089447)

Chi²: 1.19451541068775E-18 (Weighted: 0.453485509535331)

SEOE: 1.0895811558965E-09

AIC (prop.): -13587.6128666428

R^2: Nonlinear: 0.89386234455943, Linear: 0.85893292388968

Adj. R^2: Nonlinear: 0.893207173846834, Linear: 0.858497531679463

Statistics for: Elastance

SSR: 1.75892233274553E+22 (Weighted: 48.8227043421494)

Chi²: 5.42877263193066E+19 (Weighted: 0.150687359080708)

SEOE: 7345384454.44521

AIC (prop.): 14571.1275468497

R^2: Nonlinear: 0.741658400082235, Linear: 0.519918549103814

Adj. R^2: Nonlinear: 0.740063698848175, Linear: 0.518436816230677

D. Datasource: PCuT.txt_1

Model: P-(R)(C)

n Datapoints: 183 (366 total dependant values)

Free parameters: 2

DOF: 364

Last transfer function: Impedance

Last weight mode: Proportional Weighting

Fitparameters (from last fit)

CPE Q 1 : 1.050000E-008 (+- 0.000000E+000)

CPE Alpha 1 : 9.354001E-001 (+- 9.559081E-003)

Resistance 1 : 4.040000E+004 (+- 0.000000E+000)

Capacitance 1: 2.129901E-011 (+- 1.519503E-012)

Statistics for: Impedance

SSR: 504884030125.5 (Weighted: 67.574682250724)

Chi²: 1387044038.80632 (Weighted: 0.185644731458033)

SEOE: 37141.1438055484

AIC (prop.): 8132.5899909752

R^2: Nonlinear: 0.493286810526667, Linear: -0.653659402799233

Adj. R^2: Nonlinear: 0.490502672122967, Linear: -0.658202423136593

Statistics for: Admittance

SSR: 3.57964619367455E-07 (Weighted: 295.798569534935)

Chi²: 9.83419283976525E-10 (Weighted: 0.812633432788284)

SEOE: 3.12737175293856E-05

AIC (prop.): -6739.52574823053

R^2: Nonlinear: 0.950943318298911, Linear: 0.938771464479149

Adj. R^2: Nonlinear: 0.950673776091762, Linear: 0.938603254216729

Statistics for: Complex Capacitance

SSR: 2.00584659136061E-16 (Weighted: 295.798569534935)

Chi²: 5.51056755868298E-19 (Weighted: 0.812633432788284)

SEOE: 7.40300963639244E-10

AIC (prop.): -15607.149961819

R^2: Nonlinear: 0.878574552717013, Linear: 0.844586667998045

Adj. R^2: Nonlinear: 0.877907379929744, Linear: 0.844159708294743

Statistics for: Elastance

SSR: 6.34594669297272E+22 (Weighted: 67.574682250724)

Chi²: 1.74339194861888E+20 (Weighted: 0.185644731458033)

SEOE: 13167631589.087

AIC (prop.): 17000.2142045637

R^2: Nonlinear: 0.755999633055101, Linear: 0.481294483483459

Adj. R^2: Nonlinear: 0.754658971698261, Linear: 0.479869468328194

E. Datasource: PFeN.txt_1

Model: P-(R)(C)

n Datapoints: 188 (376 total dependant values)

Free parameters: 1

DOF: 375

Last transfer function: Impedance

Last weight mode: Proportional Weighting

Fitparameters (from last fit)

CPE Q 1 : 2.420000E-008 (+- 0.000000E+000)

CPE Alpha 1 : 9.010000E-001 (+- 0.000000E+000)

Resistance 1 : 3.050000E+004 (+- 0.000000E+000)

Capacitance 1: 7.907185E-011 (+- 5.346135E-012)

Statistics for: Impedance

SSR: 1816167310564.73 (Weighted: 80.8098083107461)

Chi²: 4843112828.17262 (Weighted: 0.21549282216199)

SEOE: 69499.8720067078

AIC (prop.): 8202.10320036636

R^2: Nonlinear: 0.325920331035144, Linear: -0.424941342072945

Adj. R^2: Nonlinear: 0.324122785251238, Linear: -0.424941342072945

Statistics for: Admittance

SSR: 1.92366530124152E-05 (Weighted: 1671.18289482011)

Chi²: 5.12977413664404E-08 (Weighted: 4.4564877195203)

SEOE: 0.000226188662853008

AIC (prop.): -6077.23780078279

R^2: Nonlinear: 0.693613607721712, Linear: 0.626801933250159

Adj. R^2: Nonlinear: 0.692796577342303, Linear: 0.626801933250159

Statistics for: Complex Capacitance

SSR: 9.02691689205032E-16 (Weighted: 1671.18289482011)

Chi²: 2.40717783788009E-18 (Weighted: 4.4564877195203)

SEOE: 1.54944369667385E-09

AIC (prop.): -15054.900248178

R^2: Nonlinear: 0.524008909234283, Linear: 0.415694477749725

Adj. R^2: Nonlinear: 0.522739599658908, Linear: 0.415694477749725

Statistics for: Elastance

SSR: 6.6683867475717E+21 (Weighted: 80.8098083107461)

Chi²: 1.77823646601912E+19 (Weighted: 0.21549282216199)

SEOE: 4211302783.83766

AIC (prop.): 17179.7656477616

R^2: Nonlinear: 0.747537394323177, Linear: 0.29669344246499

Adj. R^2: Nonlinear: 0.746864160708039, Linear: 0.29669344246499

F. Datasource: PFeT.txt_1

Model: P-(R)(C)

n Datapoints: 197 (394 total dependant values)

Free parameters: 1

DOF: 393

Last transfer function: Impedance

Last weight mode: Proportional Weighting

Fit parameters (from last fit)

CPE Q 1   : 1.890000E-008 (+- 0.000000E+000)

CPE Alpha 1 : 9.550000E-001 (+- 0.000000E+000)

Resistance 1 : 3.860000E+004 (+- 0.000000E+000)

Capacitance 1: 7.720566E-011 (+- 5.053254E-012)

Statistics for: Impedance

SSR: 3202817666433.76 (Weighted: 85.6549685907874)

Chi²: 8149663273.36834 (Weighted: 0.21795157402236)

SEOE: 90160.8498674266

AIC (prop.): 8754.1933557429

R²: Nonlinear: 0.293468377568009, Linear: -0.510335681691989

Adj. R²: Nonlinear: 0.291670587180141, Linear: -0.510335681691989

Statistics for: Admittance

SSR: 1.37821593943203E-05 (Weighted: 1934.62519640055)

Chi²: 3.50691078735885E-08 (Weighted: 4.92271042341108)

SEOE: 0.000187029676751003

AIC (prop.): -6484.22027138274

R²: Nonlinear: 0.844583678002602, Linear: 0.819652457795918

Adj. R²: Nonlinear: 0.844188216623474, Linear: 0.819652457795918

Statistics for: Complex Capacitance

SSR: 1.40612123603997E-15 (Weighted: 1934.62519640055)

Chi²: 3.57791663114497E-18 (Weighted: 4.92271042341108)

SEOE: 1.88913621119996E-09

AIC (prop.): -15687.4444039091

R²: Nonlinear: 0.488237273912155, Linear: 0.367334715715742

Adj. R²: Nonlinear: 0.48693507868038, Linear: 0.367334715715742

Statistics for: Elastance

SSR: 7.06176470867922E+21 (Weighted: 85.6549685907874)

Chi²: 1.79688669432041E+19 (Weighted: 0.21795157402236)

SEOE: 4233587212.03744

AIC (prop.): 17957.4174882693

R²: Nonlinear: 0.74924275620458, Linear: 0.322167482938041

Adj. R²: Nonlinear: 0.748604697060063, Linear: 0.322167482938041

Reference

S1. Alqadi, M. K.; Al-Khateeb, H. M.; Alzoubi, F. Y.; Migdadi, A. B. Synthesis and Characterization of Polymer Nanocomposite Films Based on PEO/ AgNPs-PVP: Structural, Thermal, Optical, and Electrical Properties. *Journal of Inorganic and*

Organometallic Polymers and Materials 2024, *35* (2), 1281–1291. https://doi.org/10.1007/s10904-024-03220-2

S2. Abdallah, E. M.; Qahtan, T. F.; Abdelrazek, E. M.; Asnag, G. M.; Morsi, M. A. Enhanced the Structural, Optical, Electrical and Magnetic Properties of PEO/CMC Blend Filled with Cupper Nanoparticles for Energy Storage and Magneto-Optical Devices. *Optical Materials* **2022**, *134*, 113092. https://doi.org/10.1016/j.optmat.2022.113092.

S3. da Rosa, C. G.; Sganzerla, W. G.; de Oliveira Brisola Maciel, M. V.; de Melo, A. P. Z.; da Rosa Almeida, A.; Ramos Nunes, M.; Bertoldi, F. C.; Manique Barreto, P. L. Development of Poly (Ethylene Oxide) Bioactive Nanocomposite Films Functionalized with Zein Nanoparticles. *Colloids and Surfaces A: Physicochemical and Engineering Aspects* 2020, *586*, 124268. https://doi.org/10.1016/j.colsurfa.2019.124268.

S4. Xie, K.; Shi, L. Ultrathin PEO Based Electrolyte for High Voltage Lithium Metal Batteries Enabled by Polymer Host-Plasticizer Interactions. *Journal of Energy Storage* 2023, *68*, 107640–107640. https://doi.org/10.1016/j.est.2023.107640.

S5. Nematdoust, S.; Najjar, R.; Bresser, D.; Passerini, S. Understanding the Role of Nanoparticles in PEO-Based Hybrid Polymer Electrolytes for Solid-State Lithium–Polymer Batteries. *The Journal of Physical Chemistry C* **2020**, *124* (51), 27907–27915. https://doi.org/10.1021/acs.jpcc.0c08749.

S6. Ko, W.-Y.; Lee, M.-S.; Hsu, H.-C.; Lin, K.-J. One-Pot Green Synthesis of a PEO/TCPP/$LiClO_4$ Solid Polymer Electrolyte with Improvement of Ion Transport. *The*

Journal of Physical Chemistry C 2021, *125* (42), 22960–22969. https://doi.org/10.1021/acs.jpcc.1c05376.

30. Baranowska, M.; Suliborska, K.; Chrzanowski, W.; Kusznierewicz, B.; Namieśnik, J.; Bartoszek, A. The Relationship between Standard Reduction Potentials of Catechins and Biological Activities Involved in Redox Control. *Redox Biol.* 2018, *17*, 355–366. https://doi.org/10.1016/j.redox.2018.05.005.

31. Jovanovic, S. V.; Hara, Y.; Steenken, S.; Simic, M. G. Antioxidant Potential of Theaflavins. A Pulse Radiolysis Study. *J. Am. Chem. Soc.* 1997, *119* (23), 5337–5343. https://doi.org/10.1021/ja970120f.

32. Pitre, K. S. Species Sensitive Electrochemical Method for Enrichment of Azadirachtin-A from Neem Seed. *Jordan J. Chem. (JJC)* 2021, *4* (3), 271–278.

33. Akhila, A. K.; Rani, K. J. Chemistry of the Neem Tree (Azadirachta Indica A. Juss.). *Fortschritte der Chemie Organischer Naturstoffe* 1999, 47–149. https://doi.org/10.1007/978-3-7091-6394-8_2.

34. Heřmánková, E.; Zatloukalová, M.; Biler, M.; Sokolová, R.; Bancířová, M.; Tzakos, A. G.; Křen, V.; Kuzma, M.; Trouillas, P.; Vacek, J. Redox Properties of Individual Quercetin Moieties. *Free Radic. Biol. Med.* 2019, *143*, 240–251. https://doi.org/10.1016/j.freeradbiomed.2019.08.001.

35. Ito, N.; Hideki Hakamata; Fumiyo Kusu. Simultaneous Determination of β-Sitosterol, Campesterol, Stigmasterol, and Brassicasterol in Serum by High-Performance Liquid Chromatography with Electrochemical Detection. *Anal. Methods.* 2009, *2* (2), 174–179. https://doi.org/10.1039/b9ay00195f.

****
